# Molecular signature of excessive female aggression: study of stressed mice with genetic inactivation of neuronal serotonin synthesis

**DOI:** 10.1007/s00702-023-02677-8

**Published:** 2023-08-05

**Authors:** Tatyana Strekalova, Oleg Moskvin, Aayushi Y. Jain, Nikita Gorbunov, Anna Gorlova, Daria Sadovnik, Aleksei Umriukhin, Raymond Cespuglio, Wing Shan Yu, Anna Chung Kwan Tse, Allan V. Kalueff, Klaus-Peter Lesch, Lee Wei Lim

**Affiliations:** 1 Division of Molecular Psychiatry, Center of Mental Health, University Hospital of Würzburg, Würzburg, Germany; 2 Department of Psychiatry and Neuropsychology, School for Mental Health and Neuroscience, Maastricht University, Maastricht, The Netherlands; 3 Primate Research Center, University of Wisconsin-Madison, Madison, WI USA; 4 Singapore Medical School, BluMaiden Biosciences, Singapore, Singapore; 5 Department of Biomolecular Chemistry, School of Medicine and Public Health, University of Wisconsin-Madison, Madison, WI USA; 6 Laboratory of Psychiatric Neurobiology, Institute of Molecular Medicine and Department of Normal Physiology, Sechenov Moscow State Medical University, Moscow, Russia; 7 Neuroscience Research Center of Lyon, Beliv Plateau, Claude-Bernard Lyon-1 University, Bron, France; 8 Neuromodulation Laboratory, School of Biomedical Sciences, Li Ka Shing Faculty of Medicine, The University of Hong Kong, Pok Fu Lam, Hong Kong SAR China; 9 Institute of Translational Biomedicine, St. Petersburg State University, St. Petersburg, Russia

**Keywords:** Tryptophan hydroxylase-2 (Tph2), Aggression, Serotonin, Deep sequencing (mRNAseq), Predation stress, Prefrontal cortex, Mice

## Abstract

Aggression is a complex social behavior, critically involving brain serotonin (5-HT) function. The neurobiology of female aggression remains elusive, while the incidence of its manifestations has been increasing. Yet, animal models of female aggression are scarce. We previously proposed a paradigm of female aggression in the context of gene x environment interaction where mice with partial genetic inactivation of tryptophan hydroxylase-2 (*Tph2*^+/−^ mice), a key enzyme of neuronal 5-HT synthesis, are subjected to predation stress resulting in pathological aggression. Using deep sequencing and the EBSeq method, we studied the transcriptomic signature of excessive aggression in the prefrontal cortex of female *Tph2*^+/−^ mice subjected to rat exposure stress and food deprivation. Challenged mutants, but not other groups, displayed marked aggressive behaviors. We found 26 genes with altered expression in the opposite direction between stressed groups of both *Tph2* genotypes. We identified several molecular markers, including *Dgkh, Arfgef3, Kcnh7, Grin2a, Tenm1* and *Epha6*, implicated in neurodevelopmental deficits and psychiatric conditions featuring impaired cognition and emotional dysregulation. Moreover, while 17 regulons, including several relevant to neural plasticity and function, were significantly altered in stressed mutants, no alteration in regulons was detected in stressed wildtype mice. An interplay of the uncovered pathways likely mediates partial *Tph2* inactivation in interaction with severe stress experience, thus resulting in excessive female aggression.

**Supplementary Information:**

The online version contains supplementary material available at 10.1007/s00702-023-02677-8.

## Introduction

The World Health Organization (WHO) categorizes violent behavior among the top 20 causes of disability worldwide and this is likely to increase by 2030 (Vakili et al. 2015). Recently, there have been increasing numbers of reports of females displaying aggression associated with neurodevelopmental and psychiatric disorders (Freitag et al. 2018) and rising female criminality (Denson et al. 2018). Although women are generally less aggressive than men, excessive aggression in women can be harmful, leading to devastating consequences for society, such as misconduct, family conflicts and delinquency, including severe physical injury and homicide (Xiang et al. 2019). Nevertheless, the neurobiology of aggression, particularly in females, remains elusive. This necessitates the need to understand the molecular mechanisms underpinning female aggression.

Decreased synthesis of brain serotonin (5-HT) has been demonstrated to play a critical role in pathological aggression in both sexes. The key enzyme of neuronal 5-HT synthesis is tryptophan hydroxylase-2 (TPH2) (McKinney et al. 2005). Low expression of the TPH2 can be caused by such common single nucleotide polymorphisms (SNPs) in the gene’s transcriptional control region (Herrmann et al. 2007) as c.‐1449C > A (rs7963803) and c.‐844G > T (rs4570625), -7180T > G, -7065C > T, -6526A > G, and -5806G > T. These SNP variants in TPH2 gene have been implicated in psychiatric disorders presenting with high anxiety and impulsive aggression (Rotondo et al. 1999; Carkaci-Salli et al. 2006; Oades et al. 2008; Plemenitaˇs et al. 2015; Laas et al. 2017), personality disorder (Perez-Rodriguez et al. 2010), negative emotionality (Lesch et al. 2012), depression (Wigner et al. 2018) and suicidality (Ottenhof et al. 2018). These clinical observations have been recapitulated in translational studies in mice with complete inactivation of Tph2 (Tph2 − / −), which resulted in excessive aggression regardless of sex (Gutknecht et al. 2012, 2015; Angoa-Pérez et al. 2012; Weidner et al. 2019). Notably, the experience of stress may increase the incidence of aggression (Forssman et al. 2014; Xu et al. 2016). Moreover, aggression can also be triggered per se by environmental adversities and stressors (Haller et al. 2014; Takahashi and Miczek 2013; Xiang et al. 2019; Strekalova et al. 2021a, b), which are also known to have sex-specific effects (Takahashi and Miczek, 2013; Gorlova et al. 2023). Finally, there is accumulating evidence for the relevance of gene x environment interaction in neuropsychiatric conditions in general (Lesch 2005; Caspi et al. 2002; Lesch et al. 2012; Lesch and Mössner 2006; Xiang et al. 2019; Lee et al. 2019) and in the regulation of pathological aggression, in particular, suggesting a role for genetic variation and stress factors. However, there are only a limited number of available experimental models for studying aggression in the context of gene x environment interaction (Gorlova et al. 2023). Moreover, there is a lack of models for female aggression, which limits our understanding of the molecular mechanisms.

We recently described a mouse model of female aggression, in which mice with partial genetic inactivation of *Tph2* (heterozygous *Tph2*^+/−^ mice) were subjected to predation stress, resulting in pathological aggressive behavior (Svirin et al. 2022a) reminiscent of null mutant (*Tph2*^−/−^) mice (Gutknecht et al. 2012, 2015). This was accompanied by abnormal expression of 5-HT receptors as well as changes in biomarkers of cellular distress, myelination and neural plasticity (Svirin et al. 2022a). However, these changes were not observed in naïve *Tph2*^+/−^ mice. Compared to *Tph2*^−/−^ mutants, which lack 5-HT synthesis in the brain's raphe nuclei, *Tph2*^+/−^ mice display only a 10–30% reduction of 5-HT levels (Gutknecht et al. 2012), whereas five days of rat-exposure stress induced aggressive behavior in both male and female mice that was comparable to the effects of complete absence of brain 5-HT (Gorlova et al. 2020; Strekalova et al. 2021a, b; Svirin et al. 2022a). Specifically, in the prefrontal cortex (PFC), stressed female mutants displayed altered gene expression of 5-HT receptors, *Htr1a* and *Htr2a*, glycogen synthase kinase-3β (*Gsk-3β*), *c-fos*, and myelination-related transcripts, including myelin basic protein (*Mbp*), proteolipid protein 1 (*Plp1*), myelin-associated glycoprotein (*Mag*), and myelin oligodendrocyte glycoprotein (*Mog*) as well as plasticity markers, including synaptophysin (*Syp*) and cAMP response element binding protein (*Creb*) (Svirin et al. 2022a).

In a similar paradigm of 5-day predation stress in male *Tph2*^+/−^ mice, mutants showed excessive aggressiveness with pathological behavioral patterns of impulsive aggression, which was accompanied by changes in the concentration and metabolism of 5-HT (Gorlova et al. 2020), and abnormal levels and turnover rates of dopamine and norepinephrine (Strekalova et al. 2021a, b). Remarkably, behavioral changes in male *Tph2*^+*/−*^ mice were found to be the opposite to those found in *Tph2*^+*/*+^ mice exposed to the predation, i.e., the latter display a decrease in aggressive and dominant behaviors under these conditions (Gorlova et al. 2020; Strekalova et al. 2021a, b). Besides, under challenging conditions, unlike *Tph2*^+*/*+^ mice, male Tph2^+/−^ mutants did not show stress-induced changes in the floating behavior in the forced swim test, and stressed female *Tph2*^+*/−*^ mice had unaltered signs of anxiety and increased novelty exploration (Svirin et al. 2022a). Thus, behavioral responses to various environmental challenges in *Tph2*^+*/−*^ mice of both sexes were strikingly distinct from those of wild type mice that let us to hypothesize also different transcriptomic signatures of the response to stressors of the two genotypes.

The aim of this study was to examine the potential changes of the transcriptome in the brain underlying distinct behavioral responses including excessive aggression following subchronic predation stress and food deprivation in female *Tph2*^+/−^ mice. We performed deep sequencing to investigate changes in the PFC, a region of the brain implicated in both aggression and the response to stress (Arnsten 2009; Wall et al. 2012; Takahashi et al. 2014; Achterberg et al. 2016). Previous studies showed that stressed *Tph2*^+/−^ mice displayed significant changes in the PFC including 5-HT turnover (Gorlova et al. 2020), 5-HT receptors and markers of distress, neuronal plasticity, and myelination (Svirin et al. 2022a). Furthermore, the elevated 5-HT turnover in the PFC correlated with the degree of aggressiveness in stressed male *Tph2*^+/−^ mice (Strekalova et al. 2021a, b).

In this study, we examined social/aggressive-like behavior in stressed, food-deprived female mice using previously established tests of home-cage sociability and food competition (Svirin et al. 2022a). Here, stressed *Tph2*^+/−^ female mice revealed not only increased signs of dominant and aggressive behavior, but surprisingly, also killing behavior. We investigated the molecular correlates of this behavior in the prefrontal cortex using previously developed deep sequencing and EBSeq methods, followed by a global downstream and regulon analyses using standard and custom packages/scripts. There were 26 genes that appeared to be significantly altered in opposite directions in the stressed groups of both *Tph2* genotypes. Based on previous findings reported in the literature, selected genes of interest associated with excessive aggression were further explored to establish relationships between these genes and the 5-HT system.

## Materials and methods

### Animals and housing conditions

The work was performed using three months-old female *Tph2*^+/−^ mice and their *Tph2*^+/+^ littermates. Mice were housed in standard cages in groups of three. For the predator stress test, 2.5-month-old Wistar rats were obtained from Janvier, France. All animals were maintained under controlled laboratory conditions (22 ± 1 °C, 55% humidity) under a reverse 12-h light/dark cycle (lights on at 19:00), with food and water provided ad libitum. Behavioral studies were carried out during dark phase of animals’ light cycle. Laboratory housing conditions and experimental procedures were set up in accordance with Directive 2010/63/EU using the ARRIVE guidelines (http://www.nc3rs.org.uk/arrive-guidelines). The experimental protocols were approved by the Ethical Committee of C. Bernard University of Lyon (08-09-2015-RC). All efforts were undertaken to minimize any potential discomfort to the experimental animals.

### Study design

**Fig. 1:**
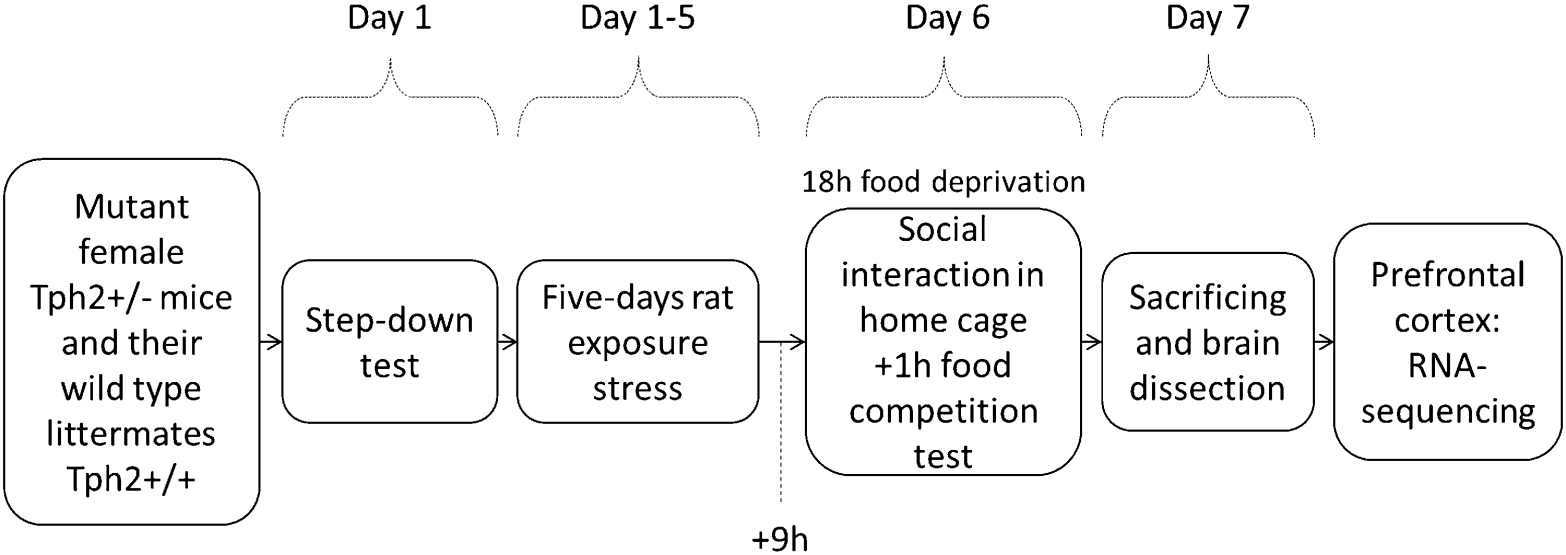
Experiment design. Female Tph2^+/−^ mice and their wildtype littermates were studied for baseline behavior in step-down test. Thereafter, they were subjected to a five-day rat exposure predation stress. In 9 h after last stress session mice were food deprived for 18 h and studied in a battery of behavioral tests for killing behavior and dominant-like behavior, then their brains were dissected for mRNA sequencing

Mutant female *Tph2*^+/−^ mice and their wildtype littermates were first assessed for baseline anxiety-like escape behaviors using the step-down test, considering these animals had no history of extensive handling; behavioral scoring was conducted on-line by manual scoring (Fig. 1). A subgroup of mice of each genotype (*n* = 9 per group) was subjected to a five-day rat exposure paradigm as described elsewhere (Vignisse et al 2017; Strekalova et al. 2021a, b; Svirin et al. 2022a). During this time period, non-stressed wildtype (*n* = 6) and non-stressed mutants (*n* = 9) were housed under standard laboratory conditions. Following nine hours recovery period after the last stress session, all mice were food deprived for 18 h and their behavior in the home cage was monitored thereafter (Fig. 1). Consequently, their interactions during the food competition test were also studied (Veniaminova et al. 2017, 2020); behavioral scoring was performed off-line, using video recordings and manual analysis. Mice were sacrificed 48 h following the end of stress procedure, their brains were dissected, the PFC was isolated for mRNA sequencing, in which brain tissue (*n* = 5) from each group) was used (Fig. 1). Bioinformatics was carried out as described elsewhere (Strekalova et al. 2015; Cline et al. 2015). The meta-analysis of the data on significantly altered gene expression was performed as described elsewhere (Strekalova et al. 2022).

### Rat exposure stress and food deprivation

Mice were introduced into a transparent glass cylinder (15 cm high × 8 cm diameter) and placed into the rat cage between 18:00 and 9:00 for five consecutive nights. Mice had free access to food and water in their home cages between the stress sessions. The timing of the rat exposure model was designed to minimize the impact of food and water deprivation, as the predation period overlaps with the light (inactive) phase of activity of the mice when food and water consumption is minimal (Strekalova 2008, 2022). A 18-h food deprivation was applied nine hours following the termination of last rat exposure stress session, as food restriction or deprivation, as a major stressor, was shown to induce agonistic behavior in mice, including females.

### Baseline escape responses and anxiety-like step-down behavior

Mice were assessed for their escape behavior during the experimental procedures. In the step-down anxiety test, the time required by the experimenter to pick up a mouse by its tail from a home cage was measured as an indirect parameter of anxiety and impulsivity (Hurst and West 2010; Mertens et al. 2019). The step-down apparatus (Evolocus LLC Tarrytown, NY, USA; Technosmart, Rome, Italy) consisted of a transparent plastic cubicle (25 × 25 × 50 cm) with a stainless-steel grid floor (33 rods, 2 mm in diameter), onto which a square wooden platform (7 × 7 × 1.5 cm) was placed. Mice were placed onto the platform inside a transparent cylinder and after removal of the cylinder, the time until the animal left the platform with all four paws was taken as a measure of anxiety (Strekalova and Steinbusch 2010; Vignisse et al. 2017). The experiment was always performed by the same person who was blinded for mouse genotype.

### Home cage interactions

Starting nine h after the termination of the last stress session, all experimental groups were food deprived for 18 h and thereafter were assessed for their social behaviors in the home cage. Surprisingly, we detected mouse killing behavior, a form of ‘intraspecific predation’ that was described earlier in mice, particularly in females with challenged 5-HT system of the brain and under conditions of stress and restricted access to the food. The incidence of killing interactions was assessed per cage and per number of animals registered prior and after food deprivation. Besides, dominant and aggressive behaviors were recorded in the home cage in all experimental groups during a 10-min period under low lighting (5 lx, white light). For behavioral analysis of dominant and aggressive interactions while in a home cage, the top of a home cage was replaced with a transparent cover, and mice were scored for previously established parameters of following and crawl-over behaviors as described elsewhere (Svirin et al. 2022a). Briefly, following behavior, a sign of hierarchical dominance in female mice was defined as the rapid chasing of a fleeing counter-partner where the maximum distance between the animals was one body length. The crawl-over behavior, also considered as a manifestation of hierarchical dominance, was defined as the movement of a mouse over the body of the partner; predominantly head, first crossing transversely from one side to the other. The number of mice expressing these behaviors was recorded. In this study, unlike male mutants, female mice of either genotype did not display signs of aggressive behavior in this test, such as fighting, biting and attacking (Gorlova et al. 2020; Strekalova et al. 2021a, b) so aggressive behavior could not be scored.

### Food competition test

Following the home cage behavioral assessment, food-deprived animals were subjected to a food competition test and their social behavior was examined during a 15-min test. Mice were food deprived prior to the test in order to potentiate their agonistic social interactions. Pairs of mice from the same experimental group but different cages, were placed in a plastic observation cage (21 × 27 × 14 cm) and allowed to compete for a piece of meat (2 g of beef) that was placed in the observation cage for 10 min under subtle lighting (light intensity: 5 lx, white light), 5 min after the onset of the test. The latency, total duration of attacking behavior, and the number of attacks were recorded during a 15 min time period. Attacking behavior was defined by the occurrence of physical attacks by one mouse against another involving kicking, wrestling, biting, and rolling over the body of the counter-partner, which was validated in previous studies on female *Tph2*^+/−^ mice (Svirin et al. 2022a).

### Brain dissection and RNA isolation

Mice were terminally anesthetized with an intraperitoneal injection of sodium pentobarbitone, and the left ventricle was perfused with 10 mL of ice-cold saline. The brains were removed and the PFC was dissected and stored at -80 °C. RNA extraction was performed as described elsewhere (Strekalova et al. 2021a, b). Briefly, total RNA was isolated from each PFC sample using TRI Reagent (Invitrogen, Carlsbad, CA, USA).

### mRNA sequencing and data analysis

Gene expression *deep sequencing* (mRNAseq) of the PFC of mice from all four groups was preformed using the BGISEQ-500 sequencing platform at BGI (Shenzhen, China). RNA quantity and quality of each sample were examined by Fragment Analyzer. RNA samples with RIN ≥ 7 and 28S/18S ratio ≥ 1 were included in the sequencing experiment. 1 µg RNA per sample was inputted for mRNA library construction followed by sequencing on the BGISEQ-500 platform. Raw reads acquired were filtered to remove reads with adaptors, reads with more than 10% unknown bases, and low-quality reads (> 50% proportion of bases with a quality score less than 5) using the SOAPnuke filtering software. Clean reads were aligned to the *Mus_musculus* genome (GCF_000001635.26_GRCm38.p6) using HISAT software (Strauss et al. 2014) and mapped to reference genes using Bowtie2 tool (Griswold et al. 2015). The gene expression level of each sample was calculated by RSEM software (Li and Dewey 2011). The count data matrix generated by RSEM (Posterior Mean Estimate version) was normalized using median normalization routine from EBSeq package (Leng et al. 2013). Differential expression was called using EBSeq with 10 iterations. Genes with assigned value of Posterior Probability of Equal Expression below 0.05 were selected as differentially expressed. Transcript Per Million (TPM) matrix generated by RSEM was used for visualization purposes.

### Global downstream data analysis

Data analysis was performed in R statistical environment (R Core Team 2021) using a combination of custom scripts and open-source packages, such as *ggplot2* described elsewhere (Wickham 2016).

#### Regulon analysis

To gain evidence of the transcription factors involved in the observed changes in gene expression, regulon analysis was performed. Regulon definitions were constructed by collecting transcription factor-target relationships from HTRIdb (Bovolenta et al. 2012) and CellNet (Cahan et al. 2014) and converting human gene identifiers to mouse homologs with Bioconductor’s biomaRt package (Durinck et al. 2009). The procedure for testing the functional enrichment was not restricted to the list of differentially expressed (DE) genes and instead, performed via genome-wide summarization of trends using parametric analysis of gene set enrichment (PAGE – Kim and Volsky 2005), implemented in Bioconductor’s piano package.

#### Statistical analysis

Analysis of behavioral data was performed using GraphPad Prism software version 8.3 (San Diego CA, USA). For normally distributed data, data were analyzed using unpaired *t*-test and two-way ANOVA followed by Tukey’s correction for pairwise comparisons. Kruskal–Wallis test was applied for a multiple group comparisons where the data were not normally distributed and was followed by Dunn’s test to perform pairwise comparisons between each independent group. Fisher's exact test was performed for the analysis of contingency tables. Statistical significance was set at *p* < 0.05. Data are presented as mean ± SEM.

## Results

### Altered baseline behaviors of female Tph2^+/−^ mice

**Fig. 2:**
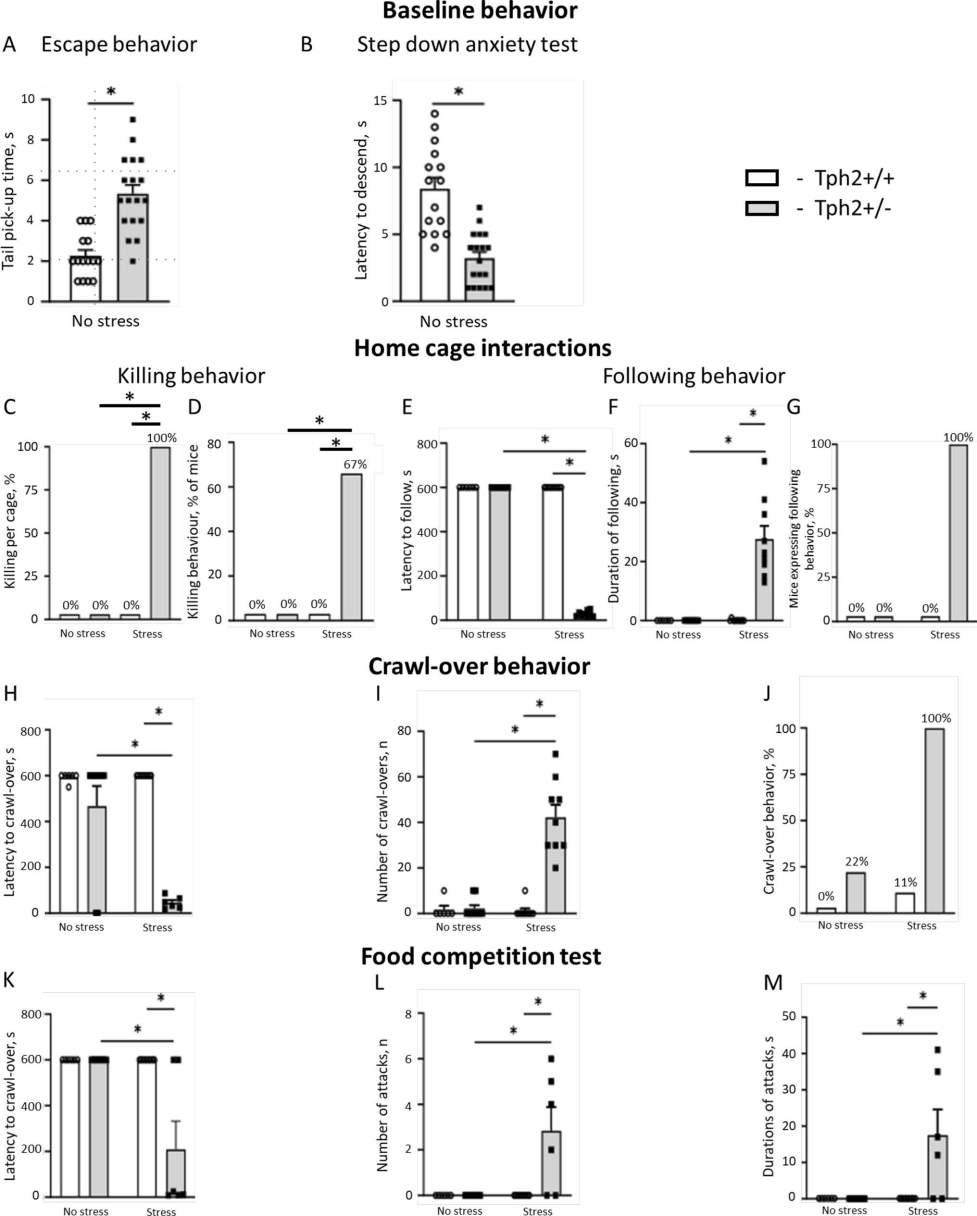
Behavioral features of naïve and stressed female Tph2 + / − mice. **A** The tail pick-up time in naïve Tph2^+/−^ mice was significantly higher than in naïve Tph2^+/+^ mice in the escape behavior assay. **B** The time to descend showed in the step-down avoidance anxiety test was decreased in naïve Tph2^+/−^ mice. **C** No significant differences were revealed in killing behavior between stressed Tph2^+/−^ group and other groups of mice taken in percent per cage. **D** Percent of mice displaying killing behavior was increased significantly in stressed Tph2^+/−^ group. **E** In the home cage observation assay, latency to follow was significantly lower in stressed Tph2^+/−^ group and **F** the duration of the following was higher in stressed Tph2^+/−^ mice. **G** The rate of mice expressing following behavior was significantly higher in stressed Tph2^+/−^ mice group. **H** Latency to crawl-over was significantly lower in the stressed Tph2^+/−^ animals and **I** the number of the crawl-overs was significantly higher in the stressed Tph2^+/−^ mice. **J** The percent of mice expressing crawl-over was higher in stressed Tph2^+/−^ mutants. **K** Latency to attack was lower in the stressed Tph2^+/−^ mice in comparison to non-stressed Tph2^+/−^ animals and stressed controls.** L** The number of episodes and **M** the duration of attacks were significantly higher in the stressed Tph2^+/−^ mice in comparison to non-stressed Tph2^+/−^ animals and stressed controls. WT, Tph2^+/+^; *p < 0.05, unpaired t-test, Kruskal–Wallis vs. and *post-hoc* Dunn’s test, *χ*^2^ and Fisher’s exact test. For statistical comparisons vs. non-stressed Tph2^+/+^ group, see Results (*not shown*). For graphs **A** and **B**, all groups, were *n* = 9, except Tph2^+/+^ non-stressed group (*n* = 6). For graphs **C-L**, stressed Tph2^+/−^ and non-stressed Tph2^+/+^ groups were *n* = 6, remaining groups were *n* = 9

In the escape behavior assay, the tail pick-up time was significantly longer in female *Tph2*^+/−^ mice than in *Tph2*^+/+^ mice (*t* = 5.120, df = 14, *p* = 0.0002, unpaired *t*-test; Fig. 2A), suggesting higher anxiety and impulsivity in the mutants. Time to descend in the step-down avoidance anxiety test was also significantly different between these groups (*t* = 5.708, df = 31, *P* < 0.0001, unpaired *t*-test; Fig. 2B), with *Tph2*^+/−^ mice showing shorter latency to step down than *Tph2*^+/+^ mice.

### Challenged Tph2^+/−^ females display pathological aggression in home cage

Taking into account the number of cages in which killing behavior was recorded, no significant differences were detected between the stressed *Tph2*^+/−^ group and other groups including stressed wildtype mice, non-stressed mutants, and non-stressed wildtype mice, while the incidence of this behavior was registered in each cage containing stressed *Tph2*^+/−^ mice (*p* = 0.1, Fisher’s exact test; Fig. 2C). I.e., killing behavior was displayed in all three cages of stressed *Tph2*^+/−^ mice after 18 h of food deprivation. A comparison of the number of mice displaying this behavior in each experimental group revealed significant differences in this measure, as six out of nine stressed mutants have survived group housing following a food deprivation (*p* = 0.0090; Fig. 2D).

In the home cage, the latency to follow and the subsequent duration, as a measure of home cage dominance, were significantly different between groups (*H* = 30.96, *p* < 0.0001 and *H* = 29.88, *p* < 0.0001, respectively, Kruskal–Wallis test; Fig. 2E, F). The percentage of mice displaying this behavior was significantly different between the groups (*χ*^2^, *p* < 0.0001; Fig. 2G). Latency to follow in stressed *Tph2*^+/−^ mice was significantly shorter compared to non-stressed *Tph2*^+/−^, non-stressed *Tph2*^+/+^ and stressed *Tph2*^+/+^ animals (both *p* < 0.0001, Dunn’s test), whereas the following duration was significantly higher in stressed mutant mice compared to non-stressed *Tph2*^+/−^, non-stressed *Tph2*^+/+^ animals and stressed controls (*p* < 0.0001, respectively, Dunn’s test).

The latency to crawl-over and the number of crawl-overs were significantly different between groups (*H* = 16.22, *p* < 0.0010 and *H* = 24.69, respectively, *p* < 0.0001, Kruskal–Wallis test; Fig. 2H, I). The percentage of mice expressing this behavior was also significantly different between groups (*χ*^2^, *p* < 0.0001; Fig. 2J). The latency to crawl-over in the stressed *Tph2*^+/−^ group was significantly shorter compared to both non-stressed *Tph2*^+/−^ and stressed *Tph2*^+/+^ animals (*p* = 0.0345 and *p* = 0.0007, respectively, Dunn’s test) as well as non-stressed *Tph2*^+/+^ mice (*p* = 0.0127, respectively, Dunn’s test). The number of crawl-over episodes was significantly increased in stressed mutant mice compared to non-stressed *Tph2*^+/−^ animals and stressed controls (*p* = 0.0005 and *p* = 0.0001, respectively), as well as non-stressed *Tph2*^+/+^ mice (*p* = 0.0014, respectively, Dunn’s test).

### Aggressive behavior of stressed Tph2^+/−^ females in the food competition test

In the food competition test, significant differences were found in the latency to attack, number of attacks, and the duration of attacks (*H* = 17.73, *p* < 0.0005, *H* = 17.73, *p* < 0.0005 and *H* = 18.38, *p* < 0.0004, respectively; Kruskal–Wallis test; Fig. 2K–M). Compared to non-stressed *Tph2*^+/−^ and stressed *Tph2*^+/+^ mice, latency to attack was significantly shorter in stressed *Tph2*^+/−^ mice (both *p* = 0.0016) and as well as non-stressed *Tph2*^+/+^ mice (*p* = 0.0052, respectively, Dunn’s test). The number of episodes and the duration of attacks were significantly higher in stressed mutant mice compared to non-stressed *Tph2*^+/−^ animals and stressed controls (all *p* = 0.0016; Fig. 2L, M) as well as non-stressed *Tph2*^+/+^ mice (*p* = 0.0052, respectively, Dunn’s test). The non-stressed mice did not exhibit following or attack behavior regardless of genotype.

### Gene expression profiling

**Fig. 3:**
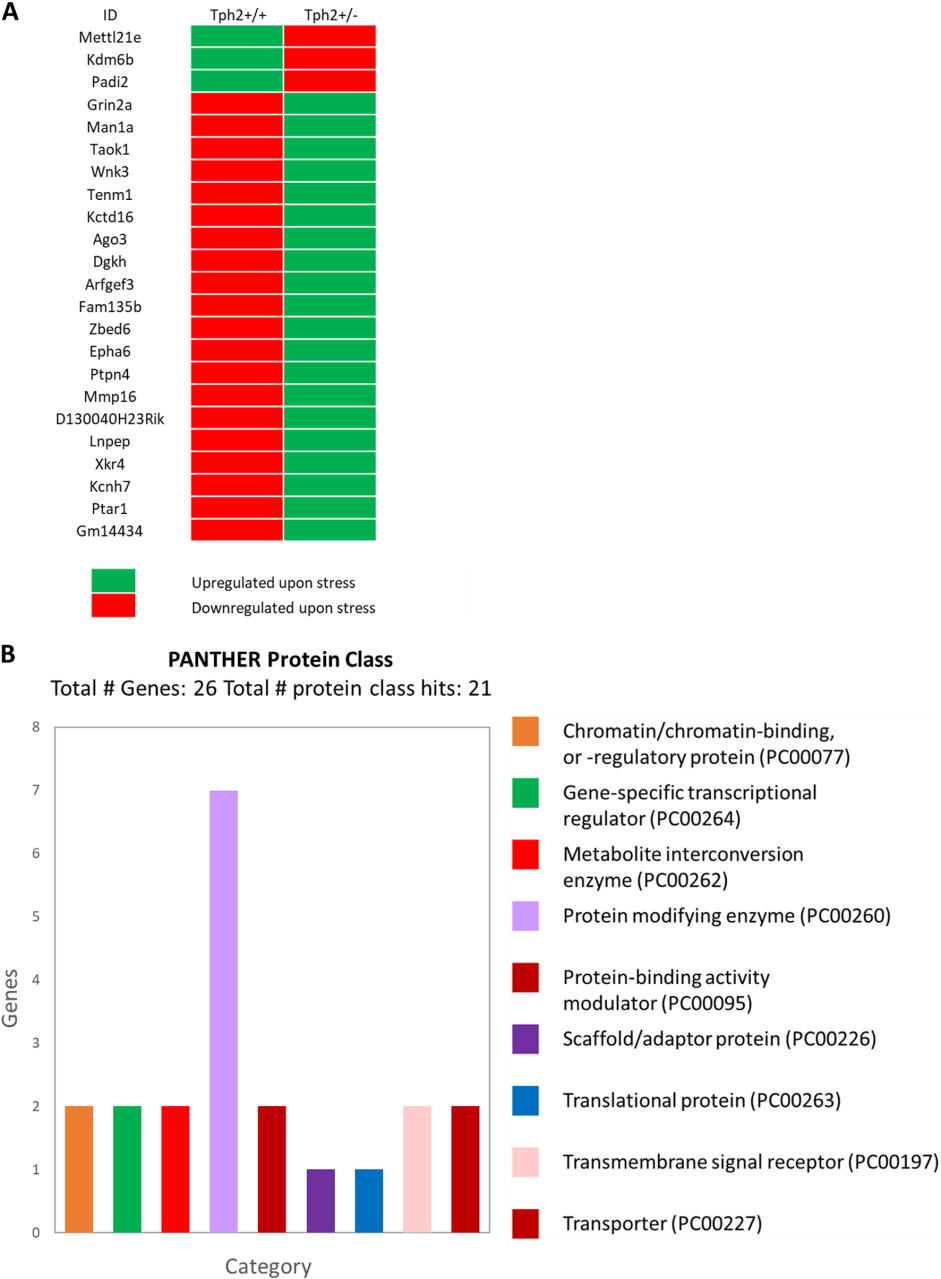
**A** Binary heatmap of directionality of gene regulation for differentially expressed (DE) genes under stress condition. Each column represents the direction of gene regulation for DE genes in wildtype and mutant under stress condition as observed for the three biological replicates from RNA-seq for each sample. **B** Classification of the DE genes using PANTHER for protein classes. Seven protein classes were identified. For all groups, *n* = 5

Differentially expressed (DE) genes were identified between wildtype *Tph2*^+/+^ mice and *Tph2*^+/−^ mutants under no-stress conditions using EBseq package based on empirical Bayes modeling for DE identification. The list of genes demonstrated differential expression between the mutant and wild type under no-stress conditions presented in *Supplementary table 2*. For this DE genes set, the direction of transcriptional regulation was identified for both *Tph2*^+/+^ and *Tph2*^+/−^ following stress experience (Fig. 3A). The nature of transcriptional regulation, i.e. upregulation or downregulation, is based on comparison of the sample under stress conditions with the respective sample under normal conditions. The change FDR of < 0.05 was applied. Gene expression profiling of experimental groups of stressed mice of the two genotypes revealed expression changes in 26 genes, in comparison to a control group under normal conditions. Among the genes upregulated in the mutants under stress conditions, including *Dgkh, Arfgef3, Kcnh7, GluN2A, Grin2a, Tenm1* and *Epha6* (*Supplementary table 1*), several have previously been implicated in neuronal function and synaptic plasticity. Correspondingly, the same genes were downregulated in wildtype mice under stress conditions (Fig. 3A). The direction of transcriptional regulation for both *Tph2*^+/+^ and *Tph2*^+/−^ are depicted in the DE gene list of Fig. 3. Like the genes implicated in neuronal function and synaptic plasticity, the other DE genes also exhibited opposite patterns of regulation for *Tph2*^+/+^ and *Tph2*^+/−^. While in *Tph2*^+/+^ most of the DE genes were downregulated under stress, the same genes were upregulated under stress condition in *Tph2*^+/−^ (Fig. 3B).

### Functional roles of differentially expressed genes

The evaluation of functional roles of DE genes was based on genes related to (a) behavioral mechanisms of aggression, (social) cognition and emotion regulation; (b) components of 5-HT system function; (c) regulation of stress responses; and (d) neurodevelopmental and psychiatric disorders (Table 1). These genes included diacylglycerol kinase eta (Dgkh), Brefeldin A-inhibited guanine nucleotide-exchange protein 3 (Arfgef3), potassium voltage-gated channel subfamily H, member 7 (Kcnh7), N-methyl-d-aspartate receptor, GluN2A subunit (Grin2a), teneurin transmembrane protein 1 (Tenm1), and ephrin-type-A receptor 6 (Epha6).

**Table 1:** Differentially expressed (DE) genes identified between wildtype TPH2 + / + mice (Wt) and TPH2 ± mutants (Mut)

ID	Gene Name	PANTHER Protein Class	Tph2 + / + foldchange	Tph2 ± foldchange	Difference Tph2 + / + vs Tph2 ±	Functional role of gene	Relation of gene to domains of interest*
Mettl21e	Protein-lysine methyltransferase METTL21E	Protein modifying enzyme	0.601	– 0.484	1.085	Maintaining of the myofiber size [1]	Not reported
Kdm6b	Lysine-specific demethylase 6B	Chromatin/chromatin-binding, or -regulatory protein	0.04	– 0.336	0.376	Regulation of synaptic plasticity and cognition [2]	Not reported
C4b	Complement C4-B	Protease inhibitor	– 0.422	– 0.32	0.102	Encodes the basic form of the complement factor 4, part of the classical activation pathway. Plays critical role in the immune system [3]	Not reported
Padi2	Protein-arginine deiminase type-2	Chromatin/chromatin-binding, or -regulatory protein	0.011	– 0.307	0.318	Participate in the mechamisms of tumors formation [4]	Not reported
Phip	PH-interacting protein		– 0.706	– 0.024	0.682	Was identified as a candidate gene for severe intellectual disability [5]	d
Per1	Period circadian protein homolog 1	Transcription cofactor	0.486	0.031	0.455	Circadian clock gene [6]	Not reported
Grin2a	Glutamate receptor ionotropic, NMDA 2A	Transmembrane signal receptor	– 1.023	0.064	1.087	Encodes subunit of the NMDA receptor, that is highly expressed in Hippocampus and neocortex. NMDA supports cognitive flexibility [7]	a, d
Man1a	Mannosyl-oligosaccharide 1,2-alpha-mannosidase IA	Protein modifying enzyme	– 0.422	0.129	0.551	The gene activity is associated with different types of cancer. Man1a is involved in cell migration, cell proliferation [8]	Not reported
Taok1	Serine/threonine-protein kinase TAO1	non-receptor Serine/threonine protein kinase	– 0.623	0.134	0.757	Was shown to be associated with the neurodevelopmental disorders; participates in the mechanisms of the cortical development [9]	d
Wnk3	Serine/threonine-protein kinase WNK3	Non-receptor serine/threonine protein kinase	– 0.524	0.161	0.685	Regulation of intracellular and extracellular Na + , Cl-, K + levels. It regulates downstream ion transporters. Alterations in the levels of the protein were associated with cerebral oschemia, epilepsy, glioma, authism, schizophrenia [10]	d
Tenm1	Teneurin-1		– 1.009	0.232	1.241	Mainly expressed during the embryonic neurodevelopment of neurons in hippocampus and amygdala. May also function as a cellular signal transducer [11]	d
Kctd16	BTB/POZ domain-containing protein KCTD16	Scaffold/adaptor protein	– 0.849	0.263	1.112	Several articles report the interaction of the Kctd16 with the GABA-B receptor [12]	Not reported
Ago3	Protein argonaute-3	Translation initiation factor	– 1.042	0.272	1.314	Member of the AGO proteins, that play role in RNA interference and participate in the mechanisms of short-interfering-RNA-silencing [13]	Not reported
Dgkh	Diacylglycerol kinase eta	Kinase	– 1.24	0.409	1.649	The diacylglycerol kinase eta is involved in the phosphorylation of diacylglycerine, that participates in the activation of protein kinase C. May contribute to the downregulation of the 5-HT2A receptor and 5-HAT transporter [14]	a, b, d
Arfgef3	Brefeldin A-inhibited guanine nucleotide-exchange protein 3	Guanyl-nucleotide exchange factor	– 0.847	0.412	1.259	Regulation of the GABA signaling in hippocampal neurons [15]	c
Fam135b	Protein FAM135B		– 0.99	0.419	1.409	Participates in the regulation of vesicular trafficking process	Not reported
Zbed6	Zinc finger BED domain-containing protein 6		– 1.043	0.441	1.484	Plays a critical role in skeletal muscle and organ growth [16]	Not reported
Epha6	Ephrin type-A receptor 6	Transmembrane signal receptor	– 0.614	0.477	1.091	Is involved in the processes of brain development and regulation of the cytoarchitecture of cortical neurons. The impairments in the contextual fear conditioning memory were reported in the model of gene's knockout [17]	c, d
Ptpn4	Tyrosine-protein phosphatase non-receptor type 4	Protein phosphatase	– 1.054	0.495	1.549	Regulates neuronal cell homeostasis; protects neurons against apoptosis [18]	d
Mmp16	Matrix metalloproteinase- 16	Metalloprotease	– 0.328	0.729	1.057	Was associated with tumors, carcinomas, cancers [19]	Not reported
D130040H23Rik	RIKEN cDNA D130040H23 gene	C2H2zinc finger transcription factor	– 0.65	0.869	1.519	Zinc finger transcriptional factor	Not reported
Lnpep	Leucyl-cystinyl aminopeptidase	Metalloprotease	– 1.204	0.967	2.171	Membrane-bound zinc-dependent metalloexopeptidase that inactivates vasopressin and oxitocin. May affect the regulation of the social behavior [20]	a, b, d
Xkr4	XK-related protein 4	Secondary carrier transporter	– 0.829	1.077	1.906	The functions of the gene are associated with addiction and substance abuse, cognitive deficits and executive functions. The gene was also associated with neuropsychiatric symptoms in McLeod Syndrome. Gene was associated with decreased total cerebellar volume [21]	d
Kcnh7	Potassium voltage-gated channel subfamily H member 7	Ion channel	– 1.313	1.308	2.621	Encodes the voltage-gated potassium channel; Inhibition leads to prolongation of plateau potentials of dopaminergic neurons [22]	a, d
Ptar1	Protein prenyltransferase alpha subunit repeat-containing 1	Acyltransferase	– 1.502	1.359	2.861	Predicted to be involved in protein prenylation	Not reported
Gm14434			– 1.383	1.657	3.04	Predicted to be involved in regulation of transcription	Not reported

Column **A**- ID of the gene; column **B**- Gene name; column **C**- class of the protein according to the PANTHER classification system; column **D**- foldchange observed in Wt animals; column **E**- foldchange observed in Mut; column **F**- absolute value of the difference in the foldchange between Wt and Mut; column **G**- functional role of gene; column **H**- Relation of gene to domains of interest*

*The domains of interest: a) behavioral mechanisms of aggression, (social) cognition and emotion regulation; b) components of 5-HT system function; c) regulation of stress responses; and d) neurodevelopmental and psychiatric disorders

### Regulon analysis

After identification of the transcription factors potentially involved in stress-induced gene expression changes we performed genome-wide regulon analysis and the results passing the false discovery rate threshold of 0.05. Genome-wide regulon analysis of the PFC of stressed *Tph2*^+/+^ mice revealed no significant changes in regulon signals, while there was a significant effect of stress resulting in upregulation of 12 regulons (*Ets1, Cdt1, Mybl2, Sap30, Zbtb20, Hmga1, Nono, Ruvbl2, Zfp3, Zfp715, Klf9* and *Atxn1*) (*Supplementary Table 3*) and downregulation of five regulons (*Sox17, Sox18, Notch1, Rbpj* and *Ifi205*) in the *Tph2*^+/−^ group (*Supplementary Table 4*).

## Discussion

Our findings demonstrate baseline differences in escape and anxiety-like behavior of female *Tph2*^+/−^ mice as well as excessive aggression and home-cage dominant behaviors in food-deprived female *Tph2*^+/−^ mice subjected to predation stress, thus resembling a behavioral profile reported for stress-naïve *Tph2*^−/−^ female mutants. Stressed wildtype controls and non-challenged, food-deprived *Tph2*^+/−^ mice did not show any of these behavioral changes. We identified 26 genes whose expression was changed in opposite direction between the stressed groups of both genotypes. Subsequent analysis of physiological functions of these genes revealed several molecular markers presumably mediating genetic brain 5-HT deficiency in interaction with adverse experience that resulted in pathological aggression of female mice. Several of the DE genes, including Dgkh, Arfgef3, Kcnh7, Grin2a, Tenm1 and Epha6 have been implicated in neurodevelopmental processes, neural plasticity, metabolic turnover, 5-HT system function and regulation of stress responses;. The molecular signature of the behavioral stress response of *Tph2*^+/−^ mice identifies risk genes in pathways leading to cognitive impairment and emotional dysregulation as well as related neuropsychiatric disorders.

Unexpectedly, food-deprived stressed *Tph2*^+/−^ female mice displayed killing behavior of their conspecifics that can be interpreted as a manifestation of extreme aggression. While according to classical ethology, killing behavior is rarely occurs among conspecifics (Lorenz 1966), adult mice and rats can display this form of excessive aggression under certain extreme conditions (Lane-Petter 1968), e.g. various forms of environmental stress (Jerussi and Hyde 1985; McCarthy and Saal 1986; Zafar et al. 2018), malnutrition and dietary restrictions (Hilakivi-Clarke et al. 1996), territorial limitations (Shimamoto 2018; Weber et al. 2017), exposure to toxins (Schardein et al. 1978; Marsman et al. 1995), disturbed 5-HT-ergic (Parmigiani and Palanza 1991) and dopaminergic (Carter et al. 2002) monoamine brain regulation. This form of behavior is also called ‘intraspecific predation’ and often involves cannibalism (Lane-Petter 1968). Reported here for the first time killing behavior of *Tph2*^+/−^ female mice suggests the role of partial genetic deficit in *Tph* combined with subchronic stress and restricted access to food in extreme female aggression.

Among the DE genes, several were previously shown to be implicated in neurodevelopmental and brain plasticity processes and associated with increased risk for emotional dysregulation. For example, DGKH encodes diacylglycerol kinase eta, which is involved in the phosphorylation of diacylglycerine (DAG). DAG participates in the activation of protein kinase C (PKC). Thus, DGKH may contribute to the downregulation of the 5-HT2A receptor and 5-HT transporter (SERT) (Anji et al. 2001). Regulation of these critical mediators of serotonergic transmission may alter levels of extracellular 5-HT and neuronal excitability. High levels of expression were observed in brain regions implicated in cognition and emotionality. While inactivation of Dgkh in mice increases levels of dopamine in the midbrain (Asami et al. 2021), variants of DGKH were associated with ADHD and bipolar disorder (Weber et al. 2011; Whalley et al. 2012). Taken together, the upregulation of Dgkh gene may contribute to the observed increased aggression and anxiety-like behavior by impacting both serotoninergic and dopaminergic transmission. Altered expression of ARFGEF3 that encodes Brefeldin A-inhibited guanine nucleotide-exchange protein 3 (BIG3) has been linked to metabolic regulation. The protein is localized in insulin- and glucagon-containing granules in pancreatic beta- and alpha-cells as well as in synaptic vesicles of neurons. Depletion of ARFGEF3 in hippocampal neurons affects lysosomal biogenesis by reducing the number of lysosomes (Liu et al. 2016). This increases levels of GABAA receptors leading to increased GABA-dependent currents and thus enhanced GABA signalling (Liu et al. 2016). Since GABA receptors have been shown to mediate fast synaptic inhibition in neural circuits (Hsu et al. 2003) implicated in the behavioral responses to stress (Paredes and Ågmo 1992), Arfgef3 upregulation may contribute to the anxiety and stress-related behavior observed in *Tph2*^*+/−*^ mice. KCNH7 encodes Kv11.3 that represents the voltage-gated potassium channel, subfamily H, member 7. The highest levels of expression are observed in limbic and cortical areas involved in the modulation of cognition and mood (Strauss et al. 2014). Dysfunction of potassium currents caused by KCNH7 inhibition leads to prolongation of plateau potentials of dopaminergic neurons. Variants of KCNH7 were associated with a wide range of neuropsychiatric disorders, including developmental delay, bipolar disorder and schizophrenic psychoses (Griswold et al. 2015). Altered plateau-potentials of dopaminergic neurons may change the release of mesolimbic dopamine, thus contributing to the abnormal behavior observed in *Tph2*^*+/−*^ mutants. GRIN2A encodes the GLuN2A subunit of N-methyl-d-aspartate (NMDA) receptors. NMDA receptors (NMDARs) are highly expressed in the hippocampus and neocortex, localize at synaptic sites and potentially interact with teneurins (Sando et al. 2019; Franchini et al. 2020). NMDARs play a critical role in modulating excitatory synaptic transmission, affecting normal neuronal development and synaptic plasticity involving long-term potentiation as well as cognition, learning and memory (Strehlow et al. 2019). Upregulation of the GRIN2A expression results in increased NMDAR-mediated currents, thus modulating excitation/inhibition (E/I) balance (van Rhijn et al. 2022). Variants in GRIN2A were associated with developmental and epileptic encephalopathy, disorders of the epilepsy aphasia spectrum, speech disorders and schizophrenic psychoses (Strehlow et al. 2019; Franchini et al. 2020; Poltavskaya et al. 2021; van Rhijn et al. 2022). Ionotropic glutamate receptors of the NMDA subtype have been implicated in cognitive flexibility. The observed upregulation of the Grin2a gene may trigger hyperactivity of the dopaminergic network, manifested by increased excitability of mutant animals (van Rhijn et al. 2022). TENM1 encodes teneurin transmembrane protein 1, which is mainly expressed during the embryonic development of neurons in hippocampus and amygdala. Protein activity promotes actin cytoskeletal remodelling and directs neuronal connectivity. In mice, it was shown that TENM1 might participate in synaptic-matching of axons of the olfactory system and inactivation of the gene in mice results in alterations associated with olfaction (Alkelai et al. 2016). Upregulation of TENM1 contributes glioblastoma cell migration and invasion of the surrounding environment (Talamillo et al. 2017). Finally, TENM1 has been implicated in X-linked intellectual disability (Bengani et al. 2021). The upregulated genes may be operational during developmental differentiation of neurons in the hippocampus and amygdala, since both brain regions are central to emotional responses, including increased impulsivity and aggression. EPHA6 encodes ephrin-type-A receptor 6, a member of the ephrin receptor family. EPHA6 is involved in processes of brain development and specifically participates in the regulation of the cytoarchitecture of cortical neurons (Das et al. 2016). EPHA6 displays high levels of expression in the hippocampus and various cortical regions. Knockout of the gene in mice results in learning impairments and memory deficits (Alkelai et al. 2016). Epha6 knockout mice display impairments in contextual fear memory (Dines and Lamprecht 2016). Disruption of the EphA/ephrin-A signaling in mice also results in a reduction of of dopaminergic neurons and reduced volume of the substantia nigra (Sieber et al. 2004). Taken together, the alteration in the Epha6 gene expression can affect cognition and memory, thus contributing to anxiety-like behavior. In addition, the fact that a number of DE genes belong to the protein and chromatin modifier class suggests that an epigenetic regulatory mechanism is involved. It might be attractive to perform mass-spectrometry on histones to identify any differences in the histone modification landscape in mutant versus wildtype mice under stress conditions. Based on mass-spectrometry results, Chromatin immunoprecipation (ChIP) may be performed on the relevant histone modifications to identify genomic loci that are being differentially regulated in the mutants under stress conditions.

mRNA expression analysis showed that 17 regulons were significantly altered in *Tph2*^+/−^ mutants exposed to predation stress, while no effects of stress were found in wildtype mice. Among altered regulons, 12 regulons were upregulated, whereas five were downregulated. A greater number of upregulated genes were reported earlier in the brain of a subgroup of C57BL6 mice with higher baseline aggression that following exposure to chronic stress displayed stress resilience by not developing the depressive-like syndrome, in contrast to low aggressive mice that were stress-susceptible (Strekalova et al. 2004, 2011). These data echo with reported phenotype of increased aggressive traits and signs of stress resilience in mice with *Tph2* genetic deficiency. Previously, behavioral and molecular findings of lower propensity to despair behavior, anxiety and other signs of maladaptive stress response demonstrated in *Tph2*^+/−^ mice (Gorlova et al. 2020; Strekalova et al. 2021a, b; Svirin et al. 2022a) and in Tph2^−/−^ mice (Savelieva et al. 2008; Weidner et al. 2019) have been demonstrated. In this connection, it has been proposed that TPH2 deficiency might have not only negative consequences been associated with pathological aggression, impulsivity and other symptoms of deurodevelopmental pathologies, but also result to beneficial increase of stress resilience (Lesch et al., 2012).

Similarly to the present and previous findings, in a recent study of the PFC transcriptome in aggressive and non-aggressive cattle breeds, a number of upregulated transcripts were more than threefold higher in aggressive breeds (Kukekova et al. 2011; Eusebi et al. 2021). This may potentially mirror the fact that aggression, as a profoundly complex behavior, is accompanied by emotional and physical invigoration, thus requiring an involvement of enhanced neuronal activity (Kukekova et al. 2020). In light of this view, the downregulation of several Sox family members, such as *Sox17* and *Sox18*, which represent transcription factors involved in diverse cellular events during development (She and Yang 2015), may suggest that these molecules mediate synaptic remodeling and neural network plasticity in aggressive animals. SOX transcription factors also impact tissue regeneration and act either as oncogenes or tumour suppressor genes, depending on the cellular environment. They are activated or inhibited through diverse genetic and epigenetic mechanisms, including DNA copy number variation, DNA methylation and abnormal microRNA expression (Grimm et al. 2020). Of note, altered expression of *Sox17* in PFC was also reported in aggressive cattle breeds (Eusebi et al. 2021). Importantly, this transcription factor is thought to be implicated in many processes that go beyond the development stage. According to the study of Lange et al. (2014), SOX17 can regulate at least 267 genes. Other genes regulated by SOX17 may be more directly involved in controlling aggressive behavior, including Monoamine Oxidase A (MAOA) that regulates aggression across spices, since SOX17 binding sites were found in its promoter region (Chen et al. 2019). Thus, SOX17 is likely to be directly implicated in the mechanisms controlling aggressive behavior.

In line with the notion that SOX transcription factors regulate neurodevelopment, other transcriptional factors with similar functions were associated with excessive aggression in *Tph2*^+/−^mutants. For instance, *Ets1* is known to regulate a number of basic biological processes in normal cells and has been linked to the regulation of immune cell function playing roles in immunity and autoimmunity (Garrett-Sinha 2013; Dhara et al. 2022). Thus, besides potential functions in synapse formation and remodeling, this factor may be implicated in increased neuroinflammatory responses also found in models of stress-induced aggression (Svirin et al. 2022b, 2022). Similarly, *Cdt1*, encoding Cdc10-dependent transcript 1 protein, which was upregulated in the PFC of stressed *Tph2*^+/−^ mice, might be involved in neurogenesis and structural and functional circuit plasticity, as it is required for the first step in DNA replication, thus being critical for genome stability and to normal development (Pozo and Cook 2016). Likewise, *Mybl2* (alias B-Myb) is a transcription factor of the MYB transcription factor family and a physiological regulator of cell cycle progression, cell differentiation and survival (Musa et al. 2017), is likely to be involved in neurogenesis and plasticity. The same holds true for *Ruvbl2*, involved in the remodeling of chromatin, DNA damage repair, and regulation of the cell cycle (Su et al. 2022), processes essential in neurogenesis and plasticity.

Remarkably, we found an upregulation of *Zbtb20* in the stressed *Tph2*^+/−^ group. ZBTB20 was shown to be critical for the developing hippocampus. Increased methylation within the gene’s coding region was associated with major depressive disorder in genome-wide MeDIP-sequencing study on 50 monozygotic twin pairs that were discordant for depression (Davies et al. 2014). Given that depression and abnormal aggression are often comorbid, altered expression of *Zbtb20* might underlie the behavioral abnormalities reported in stressed *Tph2*^+/−^ animals. Another noteworthy finding was the altered expression of the *Hmga1* regulon in stressed mutant females, since a genetic lack of HMGA1 protein was reported to adversely affect insulin receptor (INSR) expression in cells and tissues in individuals with insulin resistance and type-2 diabetes. As a matter of fact, HMGA1 is considered as a downstream nuclear target of the insulin receptor signaling pathway (Foti et al. 2005; Chiefari et al. 2012). Restoration of HMGA1 protein expression enhanced INSR gene transcription and normalized cell-surface INSR levels and insulin-binding capacity (Foti et al. 2005). Our recent work evidenced abnormal INSR expression in stressed *Tph2*^+/−^ animals and aggressive mutants with genetic deficit of Gal5 gangliosides (Svirin et al., 2022b), that is keeping with the view of critical roles of INSR signaling in brain function (Pomytkin et al. 2018). Moreover, aberrant expression of Hmga1 was found to cause insulin resistance (Foti et al. 2005). While available literature is lacking any direct indications for a relationship between food deprivation, aggression and insulin receptor functions, a demonstration of compromised secretion of hypothalamic hormones in female mice altered insulin levels that are subjected to fasting (McCosh with et al. 2019) and other indirect findings (Pomytkin et al. 2018) additionally let to hypothesize the role of insulin receptor dysregulation via HMGA1-related mechanisms in pathological aggressive behavior. As such, changes in Hmga1 and thus INSR-mediated signaling might represent a mechanism underlying excessive aggression in stressed *Tph2*^+/−^ mice.

In addition, stressed *Tph2*^+/−^ mice displayed upregulated *Nono* regulon, whose protein product has been characterized as an transcriptional regulator implicated in intellectual disability in humans as well as cognitive and affective deficits in mice (Mircsof et al. 2015). The NONO protein was shown to moderate inhibitory synapse function, by regulating synaptic mRNA transcription and gephyrin scaffold structure, both representing critical mechanisms in the functional organization of GABAergic synapses and maintenance of excitatory/inhibitory balance. As GABAergic transmission is well documented to be involved in the regulation of aggression and GABA turnover was found to be altered in *Tph2*^+/−^ mice (Strekalova et al. 2021a, b), the changes in Nono are likely to be implicated in the behavioral phenotype of stressed *Tph2*^+/−^ animals. The link to the hormonal stress response between abnormal aggression and partial 5-HT deficiency in stressed female mutants may be mediated via upregulated *Klf9*, which is a key feed-forward regulator of the transcriptomic response to glucocorticoid receptor activity (Spoerl et al., 2012; Gans et al. 2020). Given the interrelationship between 5-HT system regulation and HPA axis, function changes in KLF9-regulated binding of glucocorticoid receptors may play a major role in the mechanisms of stress-induced aggression. Moreover, we detected downgulation of *Atxn1* regulon, in which mutations cause spinocerebellar ataxia type 1, an inherited neurodegenerative disease characterized by a progressive loss of Purkinje neurons in the cerebellum. A link between altered Atxn1 expression and impulsivity as well as cognitive functioning was reported in ADHD patients (Rizzi et al. 2011) and associations between cognitive abilities and SNPs in *ATXN1* showed replicated association, but only in patient cohorts ascertained for ADHD. It might be that upregulation of *Atxn1* in the present work supports its role in impulsive behavior that overlaps with aggression. Finally, genome-wide association studies revealed a link between expression of *Notch1*, that was downregulated in stressed *Tph2*^+/−^ mutants, and the incidence of epilepsy (Huan et al. 2017) and ADHD (Alemany et al. 2016) that again might be interpreted as a manifestation of dysregulated brain inhibitory processes, a mechanism closely related to excessive aggression. Downregulation of *Rbpj*, acting as a transcriptional activator of Notch target genes further supports the involvement of Notch1-related mechanisms in abnormal aggression.

Together, within the upregulated enriched regulons in the aggressive mouse cohort, we found biological functions associated with processes, such as cellular functions, brain development and plasticity, immune responses, insulin receptor-mediated functions, GABA-related mechanisms of impulse control as well as links to neurological, neurodevelopmental and psychiatric disorders. In translational context, our findings are in line with clinical evidence that link the Tph2 gene polymorphisms, e.g., G703T to anger-related traits and the expression of anger in women (Yang et al. 2010). Other variants of the Tph2 gene were also associated with a higher incidence of anxiety disorder in women and with peripartum major depression (Lin et al. 2009; Fasching et al. 2012).

## Conclusion

Taken together, our findings reveal new molecular signatures underlying the pathological aggression of female *Tph2*^+/−^ mice exposed to stress and food deprivation. Further studies are required to shed light on the detailed mechanisms of the relationships between 5-HT deficiency and predation stress and female aggression in the context of gene × environment interaction. Our study supports the use of this mouse model of female aggression to investigate gene x environment interaction in the regulation of emotional behavior in the context of neurodevelopmental and psychiatric disorders.

## Supplementary Information

Below is the link to the electronic supplementary material.

Supplementary file1 (PDF 1057 kb)

## Data Availability

All available data is provided with the article or with attached supplementary materials.
