# Supplementary material for: Molecular signature of excessive female aggression: study of stressed mice with genetic inactivation of neuronal serotonin synthesis
Source: J Neural Transm (Vienna). 2023 Aug 5;130(9):1113–32. doi: 10.1007/s00702-023-02677-8 (PMC10460733; doi:10.1007/s00702-023-02677-8)
Supplement: Supplementary file 1 — Supplementary file1 (PDF 1057 kb) [file 702_2023_2677_MOESM1_ESM.pdf]

Supplementary materials

Supplementary Table 1

Gene Name	Annotation	Tph2$^{+/+}$ Non-Stressed	Tph2$^{+/+}$ Stressed	Tph2$^{+/-}$ Non-Stressed	Tph2$^{+/-}$ Stressed
Dgkh	Diacylglycerol kinase	4.4	1.9	1.9	2.6
Arfgef3	Brefeldin A-inhibited guanine nucleotide-exchange protein 3	8.5	4.8	4.3	6.0
Kcnh7	Potassium voltage-gated channel subfamily H, member 7	6.5	2.7	1.8	4.6
Grin2a	N-methyl-d-aspartate receptor, GluN2A subunit	11.3	5.6	6.5	6.8
Tenm1	Teneurin Transmembrane protein 1	2.2	1.1	1.0	1.2
Epha6	Ephrin-type-A receptor 6	3.1	2.1	1.9	2.8

Supplementary Table 1. Expression values of genes of interest. Transcripts Per Million (TPM) for the genes of interest are presented for the group average.

Supplementary Table 2

	PPEE		Tph2+/+ non-Stressed, average	Tph2+/- non-Stressed	Fold Change
Per1	0		38.20	56.95	1.49
Kcnh7	0		6.53	1.77	-3.70
Map7d1	1.1102E-16		149.63	170.34	1.14
Xkr4	2.0317E-13		3.11	0.92	-3.39
Nr1d1	5.7683E-12		50.92	65.44	1.29
Lnpep	3.1311E-11		5.27	1.55	-3.40
Tmbim6	8.2227E-11		101.15	116.16	1.15
C1ql3	1.2212E-10		81.71	59.15	-1.38
Dnajb5	1.674E-10		78.48	92.35	1.18
Tenm1	7.7249E-10		2.16	0.96	-2.24
Kdm6b	1.0954E-09		6.22	9.15	1.47
Fam57b	1.5958E-09		53.65	66.08	1.23
Dgkh	4.4843E-08		4.45	1.85	-2.41
Rere	5.8149E-08		41.60	50.30	1.21
Ago3	3.9085E-07		2.87	1.35	-2.12
Gpatch8	6.5271E-07		24.49	28.98	1.18
Ptpn4	1.7103E-06		6.04	2.37	-2.55
Gm14434	2.6954E-06		7.50	1.91	-3.92
Zbed6	2.7572E-06		7.75	3.96	-1.96
Brinp2	2.9588E-06		36.48	29.86	-1.22
Ripor1	4.141E-06		41.38	49.80	1.20
Pcdh19	8.7189E-06		4.07	2.89	-1.41
Prr12	1.0569E-05		16.94	21.49	1.27
Mettl21e	2.5342E-05		5.30	8.58	1.62
Map3k15	5.2754E-05		9.25	6.51	-1.42
Lrrc7	5.3676E-05		12.23	9.55	-1.28
Esyt2	7.1083E-05		7.03	5.19	-1.36
Nfix	8.3659E-05		79.35	98.99	1.25
Wnk3	9.1239E-05		3.80	2.38	-1.59
Fam208a	0.0001108		8.66	6.81	-1.27
Mmp16	0.00017247		3.59	2.09	-1.72
Mdn1	0.00025924		4.77	3.82	-1.25
Mnt	0.00026608		15.09	19.88	1.32
Hace1	0.00035857		12.91	9.68	-1.33
Zfhx2	0.00037962		18.51	23.71	1.28
Arfgef3	0.0005838		8.48	4.33	-1.96
Arhgef28	0.00081512		7.96	5.90	-1.35
Fam135b	0.00086055		4.88	2.19	-2.23
Samd4b	0.00093068		30.22	35.68	1.18
D130040H23Rik	0.00102215		1.69	0.51	-3.33
Padi2	0.00118071		4.04	5.97	1.48
Prkdc	0.00160999		2.66	1.84	-1.44
Sccpdh	0.00175819		37.39	28.18	-1.33
Taok1	0.00198775		11.13	7.25	-1.54
Man1a	0.0031724		6.34	4.34	-1.46
Grin2a	0.00319385		11.26	6.48	-1.74
Bdp1	0.00345093		5.30	3.80	-1.39
Tmem151b	0.00430989		45.05	51.05	1.13
Ptar1	0.00567552		1.86	0.51	-3.68
Macf1	0.00747561		27.78	24.24	-1.15
Phip	0.00885183		4.64	3.14	-1.48
Fbrsl1	0.01014788		20.72	25.75	1.24
Dok6	0.01045137		7.18	3.51	-2.05
Bcar1	0.01128425		19.27	23.84	1.24
Kctd16	0.01249599		10.57	6.24	-1.69
Junb	0.01288494		30.30	41.59	1.37
C4b	0.01415038		1.42	2.56	1.80
Epha6	0.01446489		3.08	1.90	-1.62
Grm5	0.01771785		22.42	16.01	-1.40
Cadm4	0.02153255		87.48	97.21	1.11
Axin2	0.0229251		9.23	12.38	1.34
Map2k1	0.0233452		102.08	84.91	-1.20
Fntb	0.02356021		11.15	14.83	1.33
Slc7a14	0.03115868		25.50	21.83	-1.17
Egr4	0.04189208		14.52	19.25	1.33
Matn2	0.04828256		11.70	8.94	-1.31

Supplementary Table 2. A comparison of gene expression data in non-stressed Tph2+/+ vs Tph2+/- mice. Gene expression data are presented in Transcripts Per Million (TPM) for genes whose expression was statistically different between non-stressed Tph2+/+ and Tph2+/- groups of mice. TPM values for respective group-level are shown as averaged TPM; signed fold change (Tph2+/+ vs. Tph2+/-) is shown as well.

Supplementary Table 3

Regulons	Stress, Tph2+/+ background	Stress, Tph2+/− background	Target's List
Cdt1	0,46	3,49	Anapc1 Anln Apex1 Arhgap11a Arhgef39 Asf1b Aspm Atic Atxn1 Aunip Aurka Aurkb Bcl2l12 Birc5 Blm Bora Brca1 Brca2 Brip1 Bub1 Bub1b Bysl 4930503L19Rik C1qbp 4833420G17Rik Cad Cbx2 Cbx7 Ccdc34 Ccna2 Ccnb1 Ccnb2 Ccne1 Ccne2 Ccnf Cct2 Cct3 Cct7 Cdc123 Cdc20 Cdc25a Cdc25c Cdc45 Cdc6 Cdca2 Cdca3 Cdca4 Cdca5 Cdca7 Cdca8 Cdk1 Cdk4 Cdkn3 Cenpa Cenpe Cenpf Cenph Cenpi Cenpk Cenpl Cenpm Cenpn Cenpw Cep55 Cep78 Chac2 Chaf1a Chaf1b Chek1 Chek2 Chrna5 Ckap2 Ckap2l Cks1b Cks2 Clspn Crebrf Cse1l Depdc1a Depdc1b Dhfr Diaph3 Dkc1 Dlgap5 Dmc1 Dnajc9 Dnmt1 Dnmt3b Donson Dscc1 Dtl Dtymk Dut E2f1 E2f7 E2f8 Ect2 Ercc6l Esco2 Espl1 Exo1 Exosc2 Ezh2 Fam64a Fam83d Fanca Fancb Fancg Fanci Fbxo5 Fbxw9 Fen1 Foxm1 Gemin4 Gins1 Gins2 Gins3 Gins4 Gmnn Gsg2 Gtse1 H2afx H2afz Haus6 Hells Hjurp Hmga1-rs1 Hmga1 Hmga2 Hmmr Hspa14 Iars Igf2bp3 Igip Iqgap3 2810417H13Rik C330027C09Rik Kif11 Kif14 Kif15 Kif18a Kif18b Kif20a Kif23 Kif2c Kif4 Kntc1 Gm10184 Kpna2 Lig3 Lmnb1 Lmnb2 Lrr1 Lrrk2 Mad2l1 Mcm10 Mcm2 Mcm3 Mcm4 Mcm5 Mcm6 Mcm7 Melk Mki67 Mnd1 Msh2 MyS2 Ncapd2 Ncapd3 Ncapg Ncapg2 Ncaph Ndc80 Neil3 Nek2 Nle1 Nme1 Nob1 Nolc1 Nono Npm3 Nudt1 Nuf2 Nup155 Nup93 Nusap1 Oip5 Orc1 Orc6 Pafah1b3 Paics Parpbp Pbk Pcna-ps2 Pcna Pdcd11 Phf19 Plcxd1 Plk1 Plk4 Poc1a Pola1 Pola2 Pold1 Pole Pole2 Polq Prc1 Prim1 Prmt5 Pttg1 Pycr1 Racgap1 Rad18 Rad51 Rad51ap1 Rad54l Ran Ranbp1 Rcc1 Recql4 Rfc2 Rfc3 Rfc4 Rfc5 Rmi2 Rnaseh2a Rpp40 Rrm1 Rrm2 Gm3086 Ruvbl1 Ruvbl2 Saal1 Sapcd2 Sars2 Shcbp1 Ska1 Ska3 Skp2 Smarca4 Smc2 Smc4 Snrpd1 Spag5 Spc24 Spc25 Srpk1 Ssrp1 Stil Suv39h1 Suv39h2 Tacc3 Tcf19 Tex10 Tfb1m Ticrr Timeless Tipin Tk1 Top2a Tpx2 Traip Trim59 Trip13 Troap Ttk Tubb5 Tyms Ube2c Ube2t Uck2 Uhrf1 Vrk1 Wdhd1 Wdr5 Whsc1 Wrap53 Xpo5 Zbtb20 Zfp64 Zfp473 Zwilch Zwint
Mybl2	0,50	3,49	Asf1b Atad3a Atxn1 Aunip Aurkb Bcl2l12 Birc5 Blm Brca1 Bub1 Bysl Cbx2 Ccnb2 Ccnf Cct7 Cdc20 Cdc25a Cdc25c Cdc45 Cdc6 Cdca2 Cdca3 Cdca5 Cdca8 Cdkn3 Cdt1 Cenpa Cenpm Chaf1a Depdc1a Dlgap5 Dscc1 E2f1 Ercc6l Espl1 Exo1 Fam64a Fanca Fen1 Gins2 Gins4 Gsg2 Gtse1 H2afx Hjurp Hmga1-rs1 Hmga1 Kif15 Kif18a Kif20a Kif2c Lmnb2 Lrr1 Lrrk2 Mcm10 Mcm2 Mcm3 Mcm5 Mcm7 Mcm8 Melk Mki67 Ncaph Nek2 Nle1 Nolc1 Oip5 Orc1 Orc6 Patz1 Pcna-ps2 Pcna Phf19 Plk1 Poc1a Pola2 Pold1 Pole Polq Pttg1 Pycr1 Rad54l Ran Rcc1 Recql4 Rnaseh2a Ruvbl2 Sapcd2 Ska3 Spc24 Spc25 Tacc3 Ticrr Tk1 Tpx2 Troap Ttk Ube2c Ube2t Whsc1 Wrap53 Zfp473 Myb
Sap30	0,08	3,49	Anln Arhgap11a Aspm Atad2 Aurka Aurkb Bcat1 Birc5 Brca1 Brca2 Brip1 Bub1 D030056L22Rik Ccna2 Ccnb1 Ccnb2 Ccne2 Ccnf Cct2 Cdc20 Cdc25a Cdc25c Cdc45 Cdc6 Cdca2 Cdca3 Cdk1 Cdk4 Cdkn3 Cenpa Cenpe Cenpf Cenpi Cenpl Cenpn Cenpw Chek1 Ckap2 Ckap2l Cks2 Clspn Cry2 Dclre1b Depdc1a Diaph3 Dlgap5 Dnajc9 Donson Dtl Dtymk E2f1 E2f7 Ect2 Emc8 Ercc6l Eri1 Exo1 Fanci Fen1 Gtse1 H2afx Haus6 Hjurp Hmmr Hspa4 Ikbip Iqgap3 2810417H13Rik Kif14 Kif18a Kif20a Kif2c Gm10184 Kpna2 Lrr1 Mad2l1 Mcm10 Mcm3 Mcm4 Melk Mki67 Mnd1 Mppe1 Mthfd1l Mthfd2 Ncapg Ncapg2 Ncaph Neil3 Nek2 Nr3c2 Nt5dc2 Nuf2 Nup50 1700123L14Rik Odf2 Orc1 Parpbp Pbk Pcna-ps2 Pcna Phf19 Plk1 Plk4 Poc1a Polr3c Prc1 Prim2 Rad18 Rcc1 Rfc3 Rpl39l Rrm1 Rrm2 Sclt1 Shcbp1 Ska1 Smc2 Smc4 Spc25 Tfdp1 Tk1 Top2a Tpx2 Trip13 Ttk Tyms Ube2t Uhrf1 Whsc1 Zbtb20 Zwilch

Zbtb20	0,02	3,49	Acly Adhfe1 Anapc16 Arhgap11a Arhgef39 Arntl2 Asf1b Aunip Aurka Aurkb Bcat1 Birc5 Bora Brca2 Brip1 Bub1 Bub1b BC055324 Cbx7 Ccna2 Ccnb1 Ccnf Cdc20 Cdc25a Cdc25c Cdc45 Cdc6 Cdca2 Cdca3 Cdca5 Cdca8 Cdk1 Cdkn3 Cdt1 Cenpa Cenpe Cenpf Cenph Cenpi Cenpk Cenpl Cenpn Cenpo Cenpw Cep55 Chek1 Chek2 Ckap2l Cks1b Cks2 Crebrf Dclre1b Depdc1a Depdc1b Dlgap5 Donson Dpp3 Dtl Ect2 Ercc6l Exo1 Fam64a Fanci Fbxo5 Fen1 Foxm1 Gins1 Gtse1 H2afx Hjurp Hmga1-rs1 Hmga1 Hmmr Hn1 Kat2b Kif11 Kif14 Kif18a Kif20a Kif2c Kif4 Klf9 Gm10184 Kpna2 Lig3 Lmnb1 Lmnb2 Mad2l1 Mastl Mcm10 Mcm3 Mcm4 Melk Mis18a Mki67 Mnd1 Mppe1 Msh2 Mxi1 Ncapg Ncapg2 Ncaph Ncbp2 Nek2 Nle1 Nme1 Npm3 Nr3c2 Nudt21 Nuf2 Nup50 1700123L14Rik Nup93 Orc1 Parpbp Plk1 Plk4 Poc1a Pole2 Prc1 Pttg1 Rad18 Rad51 Rad51ap1 Rad54l Ran Rcc1 Rfc2 Rfc3 Rfwd3 Rhno1 Rmi2 Rnaseh2a Rpl39l Rrm2 Saal1 Sap30 Ska1 Ska3 Snrpd1 Spc25 Srsf9 Top2a Tpx2 Trip13 Ttk Tubb5 Tyms Ube2c Wdhd1 Wdr5 Xpo5 Zbtb38 Zfp3 Zfp105 Zwilch Zwint
Hmga1	0,40	2,66	Asf1b Aurkb Bcl2l12 Birc5 Bub1 Bysl Cad Ccna2 Ccnb2 Ccnf Cct7 Cdc20 Cdc25a Cdc25c Cdc45 Cdc6 Cdca2 Cdca3 Cdca5 Cdca8 Cdkn3 Cdt1 Cenpa Cenpe Cenpi Cenpn Chaf1a Chaf1b Chek1 Chrna5 Crebrf Depdc1a Dlgap5 E2f1 E2f7 Emc8 Ercc6l Exo1 Fam64a Foxm1 Gtse1 H2afx Hjurp Hmga2 Hn1 Igf2bp3 Iqgap3 Kif18a Kif20a Kif2c Lig3 Lmnb2 Lrrk2 Mad2l1 Mcm10 Mcm4 Mcm7 Med20 Gm20517 Melk Mki67 MyS2 Ncaph Nek2 Nle1 Nono Nt5dc2 Nup62 Oip5 Orc1 Phf19 Plk1 Poc1a Pttg1 Pycr1 Rad54l Ran Ranbp1 Rcc1 Rnaseh2a Ruvbl2 Ska1 Ska3 Slc46a3 Snrpf Spc24 Spc25 Tacc3 Tk1 Tpx2 Trip13 Troap Ttk Tubb5 Ube2c Ube2t Uck2 Wdr5 Whsc1 Xpo5 Zbtb20 Zfp3 Zfp64 Zkscan17
Nono	0,07	2,63	Acly Apex1 Asf1b Aurkb Bcl2l12 Birc5 Bora Brca2 Brip1 Bub1 Cad Caprin1 Ccna2 Cct2 Cct7 Cdc20 Cdc25a Cdc25c Cdc45 Cdc6 Cdca2 Cdca3 Cdk1 Cdk4 Cdkn3 Cdt1 Cenpa Cenpe Cenph Cenpl Cenpm Cenpn Chek1 Chek2 Chrna5 Cks1b Ctdspl2 Depdc1a Dhx15 Dlgap5 Donson Dtl Ercc6l Ercc8 Exo1 Exosc2 Fam60a Fanci Fbl Fbxo5 Foxm1 Gabpb1 Gins4 Gstcd Gtse1 Haus6 Hells Hjurp Hmga1-rs1 Hmga1 Hnrnpc Hspa4 Igf2bp3 Igip Ilf2 Ilf3 Kif14 Kif18a Kif20a Kif2c Kntc1 Lig3 Lmnb1 Lmnb2 Lrr1 Mad2l1 Mapkapk5 Gm42878 Mcm10 Mcm2 Mcm3 Mcm4 Mcm5 Mcm7 Mdk Med20 Gm20517 Mki67 Msh2 Msh6 Ncapd2 Ncaph Ncbp1 Ncbp2 Nek2 Nfya Nob1 Nt5dc2 Nudt21 Nup50 1700123L14Rik Nup93 Odf2 Optn Orc1 Parpbp Patz1 Plk1 Plk4 Prim2 Proser1 Prpf38a Prpf4 Pttg1 Rad54l Ran Ranbp1 Rcc1 Rfc2 Rfc3 Rfc5 Rnaseh2a Rngtt Rps3 Rrm1 Gm3086 Ruvbl1 Saal1 Sec13 Set Ska1 Ska3 Skp2 Slc46a3 Smarcc1 Smc4 Snrpd1 Snrpf Spc25 Sqle Srpk1 Srsf1 Srsf2 Srsf9 Ssrp1 Supt16 Tacc3 Tk1 Tmpo Tmx1 Top2a Trp53 Tpx2 Ttk Tubb5 Tyms Ube2c Uck2 Usp10 Xpo5 Xrcc5 Zbed4 Zfp146 Zkscan17 Zwilch
Ruvbl2	0,53	2,24	Adsl Atad3a Atxn1 Bcs1l Birc5 Brca1 Bysl C1qbp Cad Cct7 Cdc20 Cdc25a Cdc25c Cdc45 Cdc6 Cdca2 Cdca3 Cdk4 Cdkn3 Cdt1 Chaf1a Crebrf Eif2s2 Ercc6l Fam64a Fbxw9 Gins2 Gtse1 Hjurp Hmga1-rs1 Hmga1 Iars Kars Kat2b Kif15 Kif20a Kif2c Lig3 Lmnb2 Lrrk2 Mcm3 Mcm7 Mki67 Mpi Mrto4 MyS2 Nle1 Nme1 Nme4 Nob1 Oip5 Pafah1b3 Phf19 Plk1 Pold1 Prmt5 Pttg1 Pycr1 Rad54l Rccd1 Recql4 Rnaseh2a Gm3086 Ruvbl1 Sars2 Ska3 Snrnp25 Spc24 Tk1 Tmem167b Tpx2 Trip13 Troap Tubb5 Ube2c Ube2t Wrap53 Xpo5 Zfp64
Zfp3	0,00	2,09	Acly Aen Arntl2 Ascc3 Bcat1 Bcl2l12 Birc5 Ccnf Cdc25a Cdc25c Cdc6 Cenpn Chek1 Ctps Donson Ercc6l Exosc2 Fen1 Foxm1 Gtse1 H2afx Hjurp Hmga1-rs1 Hmga1 Igf2bp3 Lig3 Mpp6 Mrto4 Mtap Nle1 Nme1 Nr3c2 Nup50 1700123L14Rik Orc1 Parm1 Plk1 Poc1a Prps1l3 Rad54l Ranbp1 Rcc1 Rnaseh1 Scly Skp2 Slc46a3 Snrpf Sparcl1 Sqle Tpx2 Tubb5 Txnip Wdr5 Xpo5 Yrdc Zbtb20 Zwilch

Zfp715	0,00	1,99	Abcc4 Abr Actl6a Anln Anp32b Arhgap19 Aspm Aurka Bard1 Brca1 Brip1 Bub1b 4930503L19Rik Ccdc15 Ccdc34 Ccnb1 Cdc25c Cdc7 Cdk1 Cenpe Cenpf Cenpj Cenpq Cks1b Clpx Coq3 Cyth1 Depdc1a Depdc1b Dlgap5 Gm17018 Dtl E2f8 Ect2 Elovl6 Fanci Fhl2 Gmip Hjurp Hltf Hmmr Hsd17b6 Immp1l 2810417H13Rik Kif11 Kif14 Kif15 Kif18b Kif23 Kif4 Laptm5 Litaf Myh10 Ncapg Nuf2 Oip5 Parp2 Pccb Pole2 Polq Prim1 Racgap1 Rad51 Rad51ap1 Rfc3 Rfc4 Rmi1 Rmi2 Ska2 Ska3 Smc2 Spc24 Ssx2ip Stradb Top2a Tpgs2 Tpx2 Ttk Vbp1 Whsc1 Zwint
Klf9	0,00	1,95	Alg8 Arhgef39 Aunip Bcl6 Birc5 Brip1 Bub1b 4930503L19Rik 4833420G17Rik Ccna2 Ccnb1 Cct3 Cdc20 Cdc7 Cdca5 Cdca7 Cdk1 Cenpe Cenpf Cenpk Cep55 Cep78 Chek1 Chek2 Cpeb4 Dhx33 Dlgap5 Dnmt3b Ect2 F8 Fancg Fanci Mfsd7b Foxm1 Fxyd1 Gins1 Hells Hmgn1 Hmmr Kdm5b Kif11 Kif14 Kif18b Kif20a Kif2a Kif4 Kntc1 Gm10184 Kpna2 Lig1 Mad2l1 Mcm10 Melk Mis18a Ncapg Ncapg2 Ndc80 Nuf2 Nup93 Optn Orc6 Pde4dip Pfas Plekha8 Plk4 Pop1 Ppat Prc1 Pttg1 Racgap1 Rad51ap1 Rad54l Rcc2 Rdm1 Rfc4 Rmi2 Rrm2 Ska3 Skp2 Smarca4 Srsf9 Stil Tex10 Top2a Tpx2 Trip13 Ttk Tyms Ube2c Uhrf1 Wdhd1 Xpo5 Zbtb16 Zbtb20 Zfp286 Zwilch Zwint
Atxn1	0,00	1,40	Aunip Aurkb Blm Cbx2 Cdc123 Cdc20 Cdc25a Cdc25c Cdc6 Cdca2 Cdca3 Cdca5 Cdca8 Cdt1 Cenpa Chaf1a Cks1b Cpeb2 Depdc1a Depdc1b Dlgap5 Dnmt3b Dscc1 E2f1 Ercc6l Exo1 Fam64a Fam83d Fen1 Gsg2 Gtse1 Hjurp Kat2b Kif15 Kif20a Kif2c Mcm10 Mcm2 Mcm3 Mcm7 MyS2 Ncapd3 Ncaph Ncoa1 Nek2 Nme4 Orc1 Plk1 Poc1a Pola2 Polq Prim1 Pttg1 Rad54l Recql4 Rfc3 Rfc4 Rmi2 Rnf19a Gm3086 Ruvbl1 Ruvbl2 Sapcd2 Sars2 Ska3 Ticrr Tk1 Troap Ttk Ube2c Wrap53
Ets1	0,00	3,49	Atm Bach2 Birc3 Btg1 Btla Ccdc88c Ccr7 Cd247 Cd27 Cd28 Cd3g Cd96 Clec2i Clec2g Clec2e Clec2d Clec2h Edem1 Fli1 Flt3l Foxp1 Gpr171 Gpr18 Ikzf3 Il21r Il4ra Isg20 Itk Itpkb 4930523C07Rik Klf2 Lax1 Lck Ly9 Malt1 Msn Nfatc2 Nlrc3 Nlrc5 Nlrp1a Nlrp1b Osbpl1a P2ry10 PVRIG Rac2 Rassf3 Rcan3 Rhoh Rnf213 Rogdi Scml4 Sel1l3 Sell Sept1 Sept6 Sh2d1a Sit1 AC133103.3 AC168977.2 A630001G21Rik AC125149.5 AC125149.4 AC133103.4 AC168977.1 AC133103.5 AC132444.3 Sp110 AC125149.2 AC125149.1 AC132444.5 AC133103.1 Sp140 A530032D15Rik C130026I21Rik St8sia4 Stk10 Stk17b Stk38 Stk4 Sytl3 Tagap Tap2 Gm4907 Gm4985 Tes Tgfbr2 Tifa Tigit Tmem156 Tnfrsf10b Trac Traf3ip3 Trat1 Trbc1 Trbc2 Wipf1 Yme1l1 Zap70 Zfp867 Zfp831 Nat1 Nat2 Aars Abat Abca1 Abcb7 Abcf1 Abl1 Abr Acaa1b Acaa1a Acaca Acadm Acads Acadvl Acat3 Acat2 Acly Aco1 Acox1 Acp1 Acp2 Acta2 Actb Actn4 Actl6a Actn1 Acvr1 Acvr1b Acy1 Acyp1 Acyp2 Ada Adam8 Adam10 Adar Adarb1 Adcy3 Adcy7 Add1 Add2 Add3 Plin2 Adh5 Adk Adm Adora2a Adora3 Parp1 Parp4 Adrb1 Adsl Adss Ap2a1 Ap2a2 Ap1b1 Ap2b1 Ap1g1 Aes Aga Ager Agl Gm4737 Ahcy Ahsg Aim1 Ak2 Ak4 Akt1 Akt2 Alad Alas1 Abcd1 Aldh1a1 Aldh1a7 Aldh9a1 Aldoa Aldoart1 Aldoart2 Aldoc Akr1b3 Alox5ap Alox12b Alpl Alppl2 Alpi Akp3 Amd1 Amd2 Ampd2 Ampd3 Bin1 Ang Ang2 Ang5 Ang6 Ang4 Angpt2 Ank1 Ank2 Ank3 Slc25a4 Slc25a5 Anxa1 Anxa3 Anxa4 Anxa6 Anxa11 Aoah Apaf1 Apba2 Apbb2 Apc Apeh Birc2 Birc5 Aplp1 Aplp2 Apoa1 Apoh App Aprt Fas Fasl Aqp2 Aqp3 Aqp7 Araf Arcn1 Areg Arf1 Arf3 Arf4 Arl4d Arf5 Arf6 Arg2 Rhoc Rhog Arhgap1 Arhgap4 Arhgap5 Arhgdia Arhgdib Arl1 Arnt Arntl Arrb1 Arrb2 Arsb Asah1 Asna1 Asns Zfhx3 Atf1 Atf3 Atf4 Atic Rere Atp1a1 Atp12a Atp1b1 Atp1b3 Fxyd2 Atp2a2 Atp2a3 Atp2b2 Atp2b4 Atp5a1 Atp5b Atp5c1 Atp5d Atp5e Atp5g1 Atp5g2 Atp5g3 Atp5k Atp5j Atp6v1a Atp6v1b1 Atp6v1b2 Atp6v0c Atp6v1c1 Atp6v1e1 Atp6v0b Atp6v0a1 Atp7a Atp5o Atp7b Atr Auh Aup1 B2m Bach1 Bad Bak1 Bard1 Bax Bbs1 Bbs2 Bcat1 Bcat2 Bckdha Bckdhb Ccnd1 Bcl2 Bcl2l1 Bcl2l2 Bcl3 Bcl6 Bcl7a Bcl9 Hcn2

			Bcr Bdh1 Bfsp1 Gm5096 Bhmt Bicd1 Bid Prdm1 Blk Blm Blmh Cxcr5 Bmp1 Bmp4 Bmp8b Bmp8a Bmpr2 Polr3d Bnip1 Bnip2 Bnip3 Bnip3l Bpgm Bphl Braf Brca2 Brdt Zfp36l1 Zfp36l2 Bsg Bst1 Bst2 Btd Klf5 Btf3 Bub1 Bub1b C1qbp C8b Osgin2 Znhit2 Dagla Fmnl1 Pttg1ip 1810043G02Rik Tmem50b Car4 Car11 Car12 Cacna1a Cacna1e Ddr1 Cacnb3 Cacnb4 Slc25a20 Cad Cald1 Calm1 Calu Camk4 Camk2d Camk2g Caml Canx Capg Capn1 Capns1 4933400A11Rik Capza2 Capzb Cars Casp2 Casp3 Casp6 Casp7 Casp8 Cat Runx2 Runx1 Cbfa2t3 Runx3 Cbfb Cbl Cblb Serpinh1 Cbr3 Cbs Cck Krit1 Ccna2 Ccnb1 Ccnc Ccnd2 Ccnd3 Ccne1 Ccng1 Ccng2 Ccnh Ccnt1 Ccnt2 Cd1d2 Cd1d1 Cd2 Cd3d Cd3e Cd4 Cd5 Cd6 Cd7 Cd8a Cd9 Tnfrsf8 Tnfsf8 Cd34 Cd37 Cd38 Entpd6 Cd40lg Cd44 Cd47 Cd48 Cd53 Cd59b Cd59a Cd68 Cd69 Cd70 Cd79a Lrba Cdc5l Cdc6 Cdc20 Cdc25a Cdc25b Cdc25c Cdc27 Cdc34 Cdc34b Cdc42 Cdh13 Cdh17 Cdh18 Cdk4 Cdk5 Cdk6 Cdk7 Cdk8 Cdk9 Cdkn1b Cdkn1c Cdkn2c Cdkn2d Cdkn3 Clgn Cebpb Cebpe Cebpg Cenpa Cenpe Cenpf Cetn3 Cfl1 Ctsc Rcbtb2 Chat Chd1 Chd2 Chd3 Chd4 Chek1 Foxn3 Chit1 Chm Chml Chn1 Chn2 Lyst Chrm3 Chrna1 Chrna2 Chrnb1 Chrnb3 Chuk Cirbp Cish Tbcb Ercc8 Cks2 Ap2m1 Ap1s1 Ap2s1 Clcn2 Clcn3 Clcn5 Clcn6 Clcn7 Clu Clic1 Clk1 Clk2 Clk3 Tpp1 Cln3 Cln5 Clns1a Clptm1 Clta Cltb Cltc Ccr1 Ccr1l1 Ccr4 Plk3 Cnn2 Cnp Cnr2 Col1a1 Col2a1 Col5a2 Col6a1 Col6a3 Col11a2 Copb1 Klf6 Slc31a2 Map3k8 Cox4i1 Cox5b Gm11273 Cox6a1 Cox6b1 Cox6c Cox7a2 Cox7c Cox8a Cox10 Cp Cpa1 Cpd Cpox Cpt1a Cpt2 Cr2 Crabp2 Creb1 Atf2 Crebbp Atf6b Crebl2 Crem Crip1 Crk Crkl Crmp1 Cry1 Cry2 Cryaa Cryab Crygs Csl Cs Mapk14 Cse1l Csf2 Csk Csnk1a1 Csnk1d Csnk1g2 Csnk1g3 Gm10031 Csnk2a1 Csnk2a2 Csnk2b Csrp1 Cstf1 Cstf2 Cstf3 Ctbs Ctbp1 Ctf1 Cth Ctns Ctnnb1 Ctsb Ctsd RP23-145I16.3 Ctso Cux1 Cx3cr1 Cyb5a Cyb561 Cybb Cyc1 Cyld Cyp2e1 Cyp8b1 Cyp11a1 Cyp26a1 Dab1 Dab2 Dad1 Dag1 Dgka Dapk3 Dars Daxx Dbh Dbp Dbt Dck Dct Ddb1 Ddb2 Ddc Gadd45a Ddit3 Ddost Ddx1 Ddx3x Ddx5 Dmxl1 Dhx8 Dhx9 Ddx10 Ddx11 Dhx15 Decr1 Dffa Dffb Gm9797 Timm8a1 Dfna5 Dguok Dhfr Dhodh Dhps Nqo1 Diaph1 Diaph2 Dkc1 Dlat Dld Dlg1 Dlg2 Dlg3 Dlg4 Dlst Dlx2 Dlx4 Dmbt1 Sardh Dnm1 Dmpk Dmrt1 Dmwd Dna2 Dnase1 Dnase1l3 Dync1h1 Dync1i2 Dync1li2 Dnm2 Dnmt1 Trdmt1 Dnmt3a Dnmt3b Dntt Dock2 Dock3 Dok1 Dpagt1 Dpep1 Dph1 Dph2 Dpp4 Dpp6 Dpyd Dpys Dpysl2 Dr1 Drg2 Arid3a Rcan1 Slc26a2 Dtna Hbegf Dtx1 Dtymk Dusp1 Dusp2 Dusp4 Dusp5 Dusp6 Dusp7 Dut Dvl2 Dyrk1a Tor1a E2f1 E2f2 E2f3 E2f4 E2f6 E4f1 Ebf1 Gpr183 Ece1 Ech1 Echs1 Ect2 S1pr1 Edn1 Edn2 Edn3 Phc1 Phc2 Eef1a1 Eef1b2 Eef1d Eef1g Eef2 Efna1 Efna3 Efna4 Efna5 Efnb1 Celsr3 Celsr2 Megf8 Megf9 Egr1 Egr2 Egr3 Egr4 Eif1ax Eif2b1 Eif2s3x Epha2 Eif4a1 Eif4a2 Eif4b Eif4e Eif4ebp2 Eif4g1 Eif4g2 Eif5 Eif5a Elavl1 Elf1 Elf2 Elf4 Elk3 Elk4 Emd Mark2 Emp3 Cttn Endog Eno1 Eno1b Eno2 Ensa Epas1 Epb41 Epb41l1 Stom Epha4 Ephb3 Ephb6 Cln8 Epor Eprs Nr2f6 Ercc1 Ercc2 Ercc3 Ercc4 Ercc5 Ercc6 Erf Gm10131 Erh Ern1 Fbl Esd Esr2 Esrra Etf1 Etfa Etfdh Ets1 Ets2 Etv1 Etv3 Etv4 Etv5 Etv6 Evpl Ewsr1 Ext1 Ext2 Extl2 Extl3 Eya3 Ezh2 F2rl1 F2rl2 F5 Fabp2 Fabp5 Fanca Fancc Fancd2 Fance Acsl3 Fah Ptk2b Bptf Fancf Fancg Farsa Fasn Fau Gm9843 Fbln2 Fcgr4 Fktn Fdft1 Gm43738 Fdps Fdx1 Fdxr Fech Fen1 Fer Fgf9 Fgf14 Fgfr1 Gpc5 Fgr Fh1 Fhit Fhl2 Fkbp1a Fkbp2 Fkbp3 Fkbp4 Foxc1 Foxd1 Foxj1 Foxd2 Foxo3 Flii Flna Flnb Flot2 Flt1 Flt4 Fmo4 Aff2 Fnta Fntb Fos Fosb Fosl2 Fpgs Fxn Mtor Frg1 Cenpi Fth1 Ftl1-ps1 Ftl1 Fuca1 Fuca2 Fus Fut4 Fut8 Kdsr Fyb Fyn G6pdx G6pd2 Gabpa Gabpb1 Gak Gale Galk2 Galnt1 Gm20388 Galnt2 Galt Gamt Gap43 Gars Gart Gas1 Gas6 Gas8 Gata3 Gata4 Gba Gbe1 Gcdh Gch1 Kat2a Nr6a1 Gcsh Gdf2 Gdf10 Gdi2 Gfer Gfi1 Gfpt1 Gfra2 Ggcx B4galt1 Ggt5 Gja3 Gja5 Gjb1 Gk Gclc Gclm Gldc Gle1 Glg1 Gli1 Glo1 Gls Glrx Glul Gm2a Gmds Gmfb Gna12 Gna15 Gnai1 Gnai2 Gnai3 Gnao1 Gnaq Gnas

			Gnaz Gnb1 Gnb2 Gng4 Gng7 Gngt2 Gnl1 Gns Golga1 Golga2 Golga3 Golga4 Golgb1 Got1 Got2 Sfn Gp5 Gp9 Gpd2 Gpi1 Ccr10 Gpr3 Xcr1 Gpr6 Cxcr3 Gpr12 Gpr17 Gpr39 Ffar2 Grk5 Grk6 Mknk2 Gps1 Gps2 Gpx1 Gpx2 Gpx4 Grb2 Grb7 Grb10 Rapgef1 Grid1 Grn Grin1 Grin2d Nr3c1 Arhgap35 Grm4 Cxcl2 Cxcl3 Cxcl1 Grsf1 Gsk3a Gsk3b Gspt1 Gsr Gss Gsta1 Gm3776 Gm10639 Gsta2 Gstm4 Gstz1 Msh6 Gm5435 Gtf2a1 Gtf2a2 Gtf2b Gtf2e2 Gtf2f1 Gtf2f2 Gtf2h1 Gtf2h4 Gtf2i Gtf3a Brf1 Gm29609 Gtf3c2 Gucy2c Guk1 Gusb Gyg Gypc Gucy2e Gzma Gzmc Gzmn Gzmf Gzmg Gzmd Gzmb Gzme Gzmk Gzmm H1f0 Hist1h1c Hist1h1d Hist1h1e Gm6970 Hist1h1b Hist1h2ag H2afx H2afz Hist1h2bm Habp2 Hsd17b10 Hagh Hadha Hadhb Hadh Hbq1b Hbq1a Hccs Hcfc1 Hcls1 Hcrt Htt Gm10093 Hdac1 Hdac2 Hdc Hdlbp Hells Nckap1l Hexa Nrg1 Hic1 Hif1a Hip1 Ube2k Hint1 Hivep1 Hivep2 Hk1 Hk2 Zbtb48 Mnx1 Hlf Mr1 Hlcs Hmbs Hmgb2 Hmgn1 Gm16494 Gm6594 Gm20379 Hmgn2 Gm10282 Hmgcl Hmgcr Hmgcs1 Hmga1-rs1 Hmga1 Hmox2 Nr4a1 Hnf4a Slc29a2 Hnrnpa2b1 Hnrnpab Hnrnpc Hnrnpd Hnrnpf Hnrnph1 Hnrnph2 Hnrnph3 Hnrnpk Hnrnpl Hnrnpu Tlx2 Hoxa7 Hoxb4 Hoxb7 Hoxc6 Hoxc10 Hoxd4 Hpcal1 Hpd Hpn Hps1 Hpx Hras Agfg1 Agfg2 Hrc Hrh2 Prmt2 Prmt1 Hes1 Hsbp1 Hsd11b1 Hsd17b1 Hsd17b4 Hsf1 Hsf2 Dnajb2 Dnaja1 Hspa4 Hspa5 Hspa8 Hspa9 Hspb1 Hsp90aa1 Hsp90ab1 Hspe1 Dnajb1 Foxn2 Htr5a Hus1 Iars Ica1 Icam1 Icam2 Icam4 Irf8 Id1 Id2 Id3 Ide Idh1 Idh2 Idh3a Idh3b Idh3g Idi1 Ids Idua Ifnar1 Ifnar2 Ifng Ifngr1 Ifngr2 Ifrd1 Igbp1 Igf1 Igf2r Igfbp2 Rbpj Iglc3 Iglc1 Igll1 Iglc4 Iglc2 Ik Ikbkb Il1rap Il2ra Gm20489 Il2rg Il4 Il6 Il6ra Il6st Il7r Il10 Il12b Il12rb1 Il12rb2 Il16 Foxk2 Ilf2 Impa1 Impdh2 Incenp Ing1 Ing2 Inhbc Inpp4a Inpp5a Inpp5b Inpp5d Inppl1 Insig1 Insr Eif3e Ipp Irak1 Itga6 Ireb2 Irf1 Irf2 Irf3 Irf4 Irf5 Irf7 Irs1 Itga1 Itga2 Itga4 Itga5 Itgae Itgal Itgav Itgb1 Itgb2l Itgb2 Eif6 Itgb5 Itgb6 Stt3a Itpa Itpk1 Itpr1 Itpr2 Itpr3 Ivd Jag2 Jak1 Jak2 Jak3 Jarid2 Jun Junb Jund Cd82 Kars Kcna2 Kcna6 Kcnc3 Kcnc4 Kcnh1 Kcnh2 Kcnj11 Kcnj12 Kcnj15 Kcnma1 Kcnmb1 Kcnn3 Kcnn4 Kcnq1 Kcnq2 Khk Kif2a Kif3c Kif5b Kif5c Kifc3 Klc1 Kif11 Kifc5b Kifc1 Kpna1 Kpnb1 Gm10184 Kpna2 Kpna3 Kpna4 Tnpo1 Ipo5 Kras Krt1 Krt2 Krt19 Ktn1 L1cam Aff3 Lair1 Lama3 Lama4 Lamc1 Lamp1 Lamc2 Lamp2 Rpsa Stmn1 Lasp1 Lbr Lcn2 Lcp1 Lcp2 Lct Ldha Ldhb Ldlr Letm1 Lgals1 Lgals2 Lgals3 Lgals3bp Lgals8 Lgals9 Lif Lig1 Lig3 Lig4 Lim2 Ablim1 Limk1 Limk2 Lims1 Lipa Lipc Fads1 Fads3 Llgl1 Lman1 Lmnb1 Lmo2 Prickle3 Lmx1a Lmx1b Lnpep Vwa5a AW551984 Loxl1 Lpp Lrmp Lrch4 Gm20605 Lrp2 Lrp3 Lrp6 Lrp5 Lrpap1 Lsp1 Lta4h Ltb Ltbp2 Ltbp3 Ltf Ly6e Ly6h Cd180 Lyl1 M6pr Caprin1 Nbr1 Mxd1 Mad2l1 Smad2 Smad3 Smad4 Smad5 Smad6 Smad7 Maf Magoh Man1a Man2a2 Man2c1 Man2a1 Manba Map4 Mapt Mark3 Mars Mat2a Matk Max Maz Mbd1 Mbnl1 Mbp Mc1r Mcl1 Mcm2 Mcm3 Mcm4 Mcm5 Mcm6 Mcm7 Cd46 Mdfi Mdm2 Mdm4 Me2 Mef2a Mef2d Mefv Meis2 Map3k1 Map3k3 Map3k4 Rab8a Men1 Mettl1 Mfap1b Mfap1a Mfap3 Mfge8 Mfng Mgat1 Mgat3 Mgat5 Mia2 Mgmt Mgst2 Mgst3 Mif Atxn3 Mkln1 Mlh1 Map3k9 Map3k10 Map3k11 Mllt1 Aff1 Mllt3 Mllt6 Foxo4 Nr3c2 Trpm1 Mmp3 Mmp9 Mmp15 Mmp17 Mnat1 Mnt Mocs2 Mog Mpg Mpi Mpv17 Myo1b Msh2 Msh5 Msra Msx2 Mt2 Mt3 Mtf1 Mthfd1 Mtif2 Mtm1 Myo1f Mtr Mtx1 Muc1 Muc5ac Trim37 Mut Mutyh Mvd Mvk Mxi1 Myb Mybl1 MyS2 Mycn Gadd45b Myh9 Myl4 Myl6 Myo1a Myo1c Myo1d Myo1e Myo5a Myo7a Myo9b Ppp1r12a Ppp1r12b Nab2 Naglu Hnrnpm Nap1l1 Nap1l4 Nars Nasp Nubp1 Nbn Ncbp1 Ncf4 Nck1 Ncl Ndufa1 Ndufa3 Ndufa4 Ndufa5 Ndufa7 Ndufa8 Ndufa9 Ndufa10 Ndufab1 Ndufb3 Ndufb4 Ndufb6 Ndufb7 Gm20538 Ndufb8 Ndufb9 Ndufb10 Ndufc1 Ndufc2 Ndufs2 Ndufv1 Ndufs4 Ndufs5 Ndufs6 Ndufs8 Ndufv2 Ndufv3 Drg1 Nedd4 Rpl10a Nedd9 Nek1 Nek2 Nell2 Neu1 Nf1 Nf2 Nfatc1 Nfia Nfatc3 Nfe2l1 Nfe2l2 Nfib Nfic

			Nfkb1 Nfkb2 Nfkbia Nfkbil1 Tonsl Nfrkb Nfx1 Nfya Nfyb Nfyc Ngfr Ninj1 Ninj2 Nkg7 Nktr Nkx2-2 Nmb Nmbr Nme1 Nqo2 Nnmt Nop2 Nono Nos3 Cnot2 Cnot3 Cnot4 Notch1 Notch2 Notch4 Npas1 Npas2 Nphp1 Nptx1 Slc11a2 Nras Nrf1 Nrl Nrtn Ybx1 Nthl1 Ntsr1 Nucb1 Nucb2 Numa1 Nup88 Nup98 Nr4a2 Nvl Oas2 Oas3 Oat Tbc1d25 Oaz1 Oaz2 Odc1 Odf1 Odf2 Ogdh Ogg1 Opa1 Opcml Orc2 Orc4 Orc5 Osbp Osm Oxa1l Oxct1 P2rx1 P2rx4 P2rx5 P2rx7 P4ha1 P4hb Pa2g4 Pafah1b1 Pafah1b2 Pafah1b3 Pafah2 Prdx1 Pak1 Pak2 Pam Park2 Parn Pax2 Pax6 Pax7 Pdcl Pbx1 Pbx3 Pcx Pcbp1 Pcbp2 Pcca Pccb Pcdh1 Pcdh9 Pck2 Pcm1 Pcmt1 Pcna-ps2 Pcna Pcnt Chmp1a Cdk16 Cdk17 Pcyt1a Pdcd2 Pde2a Pde4a Pde4b Pde4d Pde6d Pde6h Pde7a Pde8a Pde9a Pdgfra Pdha1 Pdhb Pdk1 Pdk2 Pdk3 Pdpk1 Pecam1 Pepd Per1 Pex6 Pex7 Pex10 Pex12 Pex13 Pex14 Pfas Pfdn1 Pfdn2 Pfdn4 Pfdn5 Pfkfb2 Gm29427 Pfkfb3 Pfkfb4 Pfkp Pfn1 Pgam1 Pgd Pggt1b Pgk1-rs7 Pgk1 Pgm2 Phb Slc25a3 Phf1 Phf2 Phka2 Phkb Phkg2 Phyh Serpinb8 Serpinb9d Serpinb9e Serpinb9f Gm11397 Serpinb9g Serpinb9 Serpinb9b Serpinb9c Piga Pigc Pigh Pik3c2a Pik3c2b Pik3c3 Pik3ca Pik3cb Pim1 Pik3cd Pik3cg Pik3r1 Pik3r2 Pi4ka Pi4kb Pin1rt1 Pin1 Pin4 Pip4k2a Pitpna Pkd1 Pkhd1 Pknox1 Pla2g1b Plaur Plcb2 Plcl1 Plcg1 Plcg2 Plec Plek Plk1 Plrg1 Plscr1 Plscr2 1700057G04Rik Plxna1 Pnoc Pml Pmm2 Pmp22 Exosc9 Exosc10 Gm42421 Pnn Ubl3 Podxl Pola1 Polb Pold1 Pold2 Pole Pole2 Polg Polh Polr2b Polr2c Polr2d Polr2e Polr2f Polr2g Polr2h Polr2k Polrmt Pomc Por Pou2af1 Pou2f1 Pou2f2 Pou5f1 Ppa1 Med1 Gm5160 Ppia Ppib Ppid Ppm1a Ppm1g Ppox Ppp1ca Ppp1cb Ppp1cc Ppp1r2 Ppp1r7 Ppp1r8 Ppp1r10 Ppp2ca Ppp2cb Ppp2r1a Ppp2r1b Ppp2r2a Ppp2r2b Ppp2r2c Ppp2r5a Ppp2r5b Ppp2r5c Ppp2r5d Ppp2r5e Ppp3ca Ppp4c Ppp3cb Ppp3r1 Ppp5c Ppp6c Ppt1 Prcc Prcp Prep Prf1 Prim1 Prim2 Prkaa1 Prkab1 Prkab2 Prkacb Prkaca Pkia Pkib Prkag1 Prkar1a Prkar2a Prkca Prkcb Prkcd Prkce Prkcg Prkch Prkci Pkn1 Pkn2 Prkcq Prkcsh Prkcz Mapk1 Mapk6 Mapk7 Mapk13 Map2k1 Map2k2 Map2k3 Map2k5 Map2k6 Map2k7 Dnajc3 Prkx Prl Prnp Proc Pros1 Prox1 Prps1l3 Prpsap1 Prpsap2 Lgmn Try4 Try5 Try10 Prss3 Gm5771 Gm10334 Prss1 Prss2 Prss8 Psap Psen1 Psen2 Pskh1 Psma1 Psma2 Psma3 Psma4 Psma5 Psma6 Psma7 Psmb2 Gm4950 Psmb3 Psmb4 Psmb5 Psmb6 Psmb7 Psmb8 Psmb9 Psmb10 Psmc1 Psmc2 Psmc3 Psmc4 Psmc5 Psmc6 Psmd1 Gm21972 Psmd2 Psmd3 Psmd4 Psmd7 Psmd8 Psmd9 Psmd11 Psmd12 Psmd13 Psme1 Psph Ptafr Ptbp1 Ptch1 Pten Ptgdr Ptgds Ptger4 Ptgs2 Ptk2 Ptk6 Ptk7 Twf1 Ptma Qsox1 Ptpn1 Ptpn2 Ptpn4 Ptpn6 Ptpn7 Ptpn11 Ptpn14 Ptpra Ptprc Ptprcap Ptpre Ptprk Ptprn Pts Rad1 Pura Purb Pwp2 Pex19 Abcd3 Abcd4 Pex2 Pxn Pex5 Pygb Pygm Qars Qdpr Rab1a Rab2a Rgl2 Rab4a Rab5a Rab5b Rab6a Map4k2 Rab27a Rabggta Rabggtb Rabif Rab5c Rac1 Rac3 Rad9a Rad21 Rad23a Rad23b Rad51 Rad51c Rad51b Rad51d Rad52 Raf1 Rala Ralb Ralgds Ran Ranbp2 Rap1a Rap1b Rap1gap Rap1gds1 Rap2a Rap2b Rara Rarg Rars Rasa1 Rasa2 Rasgrf2 Rb1 Arid4a Rbbp5 Rbbp6 Rbbp8 Rbl1 Rbl2 Rbm3 Rbms1 Rcn2 Recql Rel Rela Upf1 Dpf2 Rev3l Rfc1 Rfc2 Rfc3 Rfc4 Rfc5 Trim27 Rfx1 Rfx2 Rfx3 Rfx5 Rfxap Rgr Rgs1 Rgs2 Rgs3 Rgs10 Rgs12 Rgs16 Rhd Rheb Ring1 Rit1 Rlf Rnase2b Ear14 Rnase2a Ear1 Ear10 Ear2 Ear6 Rnase6 Rnasel Rnf2 Brd2 Rnf4 Rnf6 Rnh1 Rock1 Rora Rorb Rorc Rp9 Rpgr Rpa1 Rpa2 Rpa3 Rpe Rpl3 Rpl4 Rpl5 Rpl6l Rpl6 Rpl8 Gm5093 Gm10036 Rpl11 Rpl12 Rpl13-ps3 Rpl13 Rpl19 Rpl22 Rpl23a-ps3 Rpl23a Mrpl23 Rpl24 Rpl26 Rpl27-ps3 Rpl30 Rpl27a Rpl28 Gm3550 Gm5218 Gm17669 Rpl29 Rpl32 Rpl34 Gm6525 Rpl37 Rpl37a Rpl38 Rpl39 Gm10076 Rpl41 Rpl36al Rplp0 Gm10073 Rplp1 Rplp2 Mrpl12 Rpn1 Rpn2 Rps3 Rps3a1 Rps4x Rps5 Rps6 Rps6ka1 Rps6ka2 Rps6ka3 Rps6kb2 Rps7 Rps8 Rps9 Rps11 Rps12-ps3 Rps12 Rps13 Rps14 Gm4775 Rps15a Rps16 Rps17 Rps19 Rps20 Rps21

			Rps23 Rps24 Rps25 Rps26 Rps27 Rps27a Gm10126 Rps29 Rras Rrbp1 Rreb1 Rrm1 Rrm2 Rs1 Clip1 Rsu1 Rtn2 Rxra Rxrb Ryr3 S100a5 S100a10 S100a11 S100b Vps52 Sars Sat1 Satb1 Msmo1 Atxn1 Atxn2 Atxn7 Scd2 Scd3 Scd1 Scd4 Clec11a Scn2a Scn2b Scn8a Sco1 Scp2 Srl Ccl3 Sdcbp Sdf2 Sdhb Sdhd Sec13 Trappc2 0610009B22Rik Sel1l Selplg Set Setmar Sfpq Sfrp2 Srsf1 Srsf2 Srsf3 Srsf4 Srsf5 Srsf6 Srsf7 Sfswap Tra2b Sgca Sgcb Sgk1 Sgsh Sgta Sh3bgr Sh3bgrl Sh3bp2 Sh3gl1 Shb Shc1 Fbxw4 Shh Shmt1 Shmt2 Shox2 Siah1b Siah1a Siah2 St6gal1 St3gal1 St3gal2 St3gal4 St3gal3 Stil Sipa1 Ski Skp1a Skp2 Sla Slamf1 Slc4a3 Slc1a4 Slc1a5 Slc2a1 Slc2a3 Slc2a4 Slc3a1 Slc3a2 Slc4a1 Smtn Slc5a3 Slc6a6 Slc6a9 Slc8a2 Slc9a1 Slc12a2 Slc14a1 Slc16a1 Slc34a1 Slc19a1 Slc20a1 Slc25a1 Slc22a4 Slc22a5 Slc22a21 Slit1 Snai2 Smarca2 Hltf Smarca4 Smarcb1 Smarcc1 Smarcc2 Smarcd1 Smarcd2 Smarce1 Smpd1 Smpd2 Sumo2 Snapc1 Snapc2 Snapc3 Snapc4 Fscn1 Snrnp70 Snrpa Gm5145 Snrpa1 Snrpb Snrpb2 Snrpc Snrpd1 Gm5449 Snrpd2 Snrpd3 Snrpe Snrpf Sntb1 Snx1 Snx2 Sntb2 Soat1 Sod1 Sord Sorl1 Sos1 Sox1 Sox4 Sox9 Sp1 Sp2 Sp3 Sp4 Spag1 Spast Spg7 Spink2 Spn Spp2 Spr Sptan1 Sptbn1 Sptbn2 Sqle Sri Srebf1 Srf Srm Srp9 Srp14 Srp19 Srp54a Srp54b Srp68 Srp72 Srpk1 Srpk2 Trim21 Ssb Ssbp1 Ssr1 Ssr2 Ssr3 Ssrp1 Sstr2 Sstr3 Ss18 St13 St14 Stat1 Stat2 Stat3 Stat4 Stat5a Stat5b Stat6 Stau1 Hspa13 Elovl4 Stim1 Nek4 Cdkl5 Stk11 Aurkc Strn Stx1a Stx3 Stx4a Stx5a Stxbp2 Stxbp3 Sult1a1 Supt4a Supt5 Supv3l1 Surf2 Surf4 Surf6 Svil Vamp1 Vamp2 Xcl1 Syk Syn1 Syp Sypl Syt5 Tacr2 Tac2 Tacc1 Adam17 Tada2a Taf1 Taf2 Taf4 Taf4b Taf5 Taf7 Ak6 Taf9 Gm4799 Taf10 Taf12 Taf13 Map3k7 Tal1 Taldo1 Tap1 Tapbp Tarbp1 Tarbp2 Tars Tat Taz Tbca Tbcc Tbcd Tbce Tbp Tbx6 Tbxa2r Tcea1 Tcea2 Tcea3 Tceb1 Tceb2 Tceb3 Tcf4 Tcf3 Tcf7 Tcf7l2 Tcf12 Zfp354a Tcf19 Vps72 Mlx Tcof1 Tcp1 Tectb Tcta Ppp1r11 Dynlt1f Dynlt1b Dynlt1a Dynlt1c Tdg Gm5225 Prdx2 Tmbim6 Tep1 Terf1 Terf2 Tert Tesk1 Tfam Tfap4 Tfcp2 Nr2f1 Nr2f2 Tfdp1 Tfe3 Tfrc Tgfa Tgfb1 Tgfb3 Tgfbi Tgfbr1 Tgfbr3 Tgif1 Thbs1 Thbs2 Thbs4 Thop1 Thra Klf10 Tia1 Tial1 Tiam1 Timp3 Tjp1 Tk2 Tkt Tle1 Tle2 Tle3 Tle4 Tln1 Tlr4 Tspan4 Tm7sf2 Trappc10 Tmf1 Tmpo Tmsb4x Clec3b Tnf Tnfaip3 Tnfrsf1a Tnfrsf1b Tns1 Top1 Top2a Top2b Trp53bp1 Trp53bp2 Tpd52 Tpd52l1 Tpd52l2 Tpm3-rs7 Tpm3 Tpm4 Tpmt Tpo Tpp2 Tpr Nr2c1 Nr2c2 Hsp90b1 Traf1 Traf2 Traf5 Traf6 Cct3 Trpm2 Tsn Tsc1 Tsg101 Tspyl1 Tssc1 Tsta3 Ttc1 Dnajc7 Ttc3 Ttc4 Ttf1 Ttk Tuba4a Tubg1 Tufm Tulp1 Tulp3 Hira Tnfsf4 Tnfrsf4 Txk Txnrd1 Tyk2 Tyms U2af1 Uba52 Ubb Uba1 Ube2a Ube2b Ube2d1 Ube2d2a Ube2d3 Ube2g1 Ube2g2 Ube2h Ube2i Ube2l3 Ube2v1 Ube2v2 Ube3a Sumo1 Ubp1 Ubtf Uchl3 Uchl4 Ucp2 Ucp3 Ufd1l Slc35a2 Ugcg Ugdh Ugp2 Uck2 Umps Ung Usp4 Nr1h2 Upk2 Uqcrb Uqcrc1 Uqcrc2 Uqcrfs1 Urod Uros Usf1 Usp1 Utrn Kdm6a Uvrag Vars Vasp Vav1 Vbp1 Vcl Vcp Vdac1 Vdac2 Vdac3-ps1 Vdac3 Vegfa Ezr Vim Vipr1 Vipr2 Vpreb2 Vpreb1 Vrk1 Vwf Wars Was Eif4h Wee1 Wfs1 Wnt7a Wnt11 Wnt2b Wrb Wt1 Xbp1 Xpa Xpc Xpnpep1 Xpo1 Xrcc1 Xrcc2 Xrcc3 Xrcc4 Xrcc5 Yes1 Yy1 Ywhab Ywhae Ywhag Ywhah Sf1 Zfp36 Zfy2 Zfx Zfy1 Zfp661 Zfp113 Zfp7 Zfp128 Cnbp Zfp12 Zkscan6 Zfp182 Zfp422 Zfp612 Zfp24 Zscan20 Zfp637 Zkscan1 Zscan21 Mzf1 D3Ertd254e Zfp94 Zbtb25 Zfp523 Zfp131 Zfp458 Zfp72 Zfp708 Gm28557 Zfp457 Zfp87 Zfp738 Zfp65 Zfp58 Zfp595 Zfp85 Zfp493 Zfp273 Zfp142 Zfp143 Zbtb16 Trim25 Zfp148 Zbtb17 Vezf1 Trim26 Zfp658 Zfp719 Zfp819 Zfp715 Zfp180 Zfp185 Zmym2 Zfp202 Zfp13 Zfp207 Zfp213 Zfand5 Zfp217 Zfp236 Slc30a2 Zp3 Map3k12 Zyx Luzp1 Prdm2 Ptp4a1 Lrp8 Laptm5 Csde1 Btg2 Alms1 Mogs Tuba1a Cxcr4 Brpf1 Ifrd2 Mapkapk3 Slmap Manf Usp7 Rab7 Slbp Dek Aldh5a1 Prrc2a Bag6 Abhd16a H2-Ke6 Lst1 Tfeb Epm2a Zfp212 Ubxn8 Kat6a Nr4a3 Brd3 Nup214 Lhx3 Stam Mllt10 Cubn Ccdc6 Gm6768 Ncoa4 Slc25a16 Rassf7 Cul5 Ptp4a2 Usp5 Mlf2 Aaas Fxr1

			Yeats4 Ift88 Gm20521 Pabpn1 Dpf3 Gpr68 Ap3b2 Anp32a Nprl3 Gan Slc7a5 Taf15 Coil Akap1 Madcam1 Sf3a2 Ell Sympk Clpp Dpf1 Ncoa3 Chaf1b D10Jhu81e Dgcr6 Lztr1 Dgcr14 Zrsr2 Zrsr1 Usp11 Usp9x Kdm5c Smc1a Lage3 Slc10a3 Tktl1 Arid1a Hist1h4f Trrap Picalm Snn Acox3 Axin1 Axin2 Bap1 Cdc7 Eomes Fzd1 Hist2h2aa2 Hist1h2bg Hist1h3a Hist1h3i Hist1h4k Hist1h4j Hist1h4b Mad1l1 Olfr412 Pip5k1a Pip4k2b Pla2g6 Spop Uxt Eea1 Ltbp4 Zfp282 Stk24 Reck Rad54l Nsmaf Nck2 Gnpat Dyrk2 Dusp11 Doc2a Dhx16 Cul4b Cul4a Cul3 Cul2 Cul1 Atrn Ttf2 Tpst2 Tpst1 Supt3 Smarca5 Ogt Gpr65 Rae1 Sema7a Ppm1d Ppfibp2 Ppfibp1 Ranbp3 Ppfia1 Slc43a1 Pik3r3 Pex3 Cntnap1 Enc1 Nipsnap1 Ndst2 Kcnab2 Ikbkap Hat1 Gcm1 Dgkz Dgke Dgkd Cst7 Cops3 Camk1 Api5 Agps Apol9a Apol9b Apol11a Apol10a Apol10b Apol11b Lmo4 Cggbp1 Ap3b1 Blzf1 Lgr5 Mapkapk5 Gm42878 Bhlhe40 Pias1 Cdc14a Cdk10 Prpf18 9130409I23Rik Degs1 Denr Thoc5 Yars Madd Rrp1 Mknk1 Khsrp Prkra Stk16 Nop14 Slc25a12 Gm3086 Ruvbl1 Klf7 Stc2 Uso1 Cdk13 Pde8b Psmg1 Rfxank Jrk Skap1 Rnaset2b Rnaset2a Ssna1 Eif4ebp3 Kcnk5 Ncoa1 Lamtor3 Numb Socs1 BC048507 Dynll1 Tnks Aldh4a1 Eif3a Eif3b Eif3d Eif3f Eif3g Eif3h Eif3i Eif3j1 Eif3j2 Eif4g3 Vamp4 Stx11 Becn1 Srsf9 Krt36 Hyal2 Dgat1 Cdc23 B4galt3 B3galt4 Mbtps1 Edf1 Snx3 Eed Ctnnal1 Adam19 Gbf1 Rnmt Rngtt Gpaa1 Myom1 Ripk1 Cradd Hrk Tnfsf14 Tnfsf10 Tnfsf9 Cds2 Gm10110 Pabpc4 Cd164 Tnfrsf14 Rab11a Ripk2 Fadd Snap23 Napg Napa Mtmr1 Riok3 Rgs9 Dyrk4 Gm42957 Pex11b Suclg2 Suclg1 Sucla2 Trim24 Ccnk Gm20716 Dpm1 Cdkl1 Dcaf5 Dpm2 Sap30 Fgf17 Ces2a Ces2b Ces2c Ces2e Ces2f Ces2g Ces2h Iqgap1 Nrp2 Nrp1 Syngap1 Cd84 Tmem11 Ggh Cflar Wisp3 Wisp2 Wisp1 Hdac3 Ksr1 Tsc22d1 Kat2b Cdk5r1 Akap4 Fcgbp 9530053A07Rik Ldb1 Per2 Synj1 St3gal5 Ier3 Synj2 Cdc123 Arhgef7 Sgpl1 Fubp1 Cdc16 Nae1 Slc5a6 Ddx18 Tax1bp1 Eif2b4 Eif2b3 Eif2b2 Eif2b5 Eif2s2 Bud31 Mtmr2 Prpf4b Cpne1 Ap1s2 Ap1g2 Ap1m1 Cacna1i Timeless Bcl10 Herc3 Herc1 Gm38393 Snurf Bsn Foxh1 Mbd4 Mbd2 Wasf1 Baiap3 Fubp3 Top3b Cdk5r2 Ap3d1 Btrc Hist2h2be H1fx P4ha2 Wasl Plod3 Rps6ka4 Stbd1 Atp6v0e Limd1 Tnfsf18 Hap1 Taf1c Taf1b Taf1a Slc25a14 Mpzl1 Map3k14 Socs3 Ch25h Rnf8 Gm28043 Hip1r Baz1b Ube2m Spag9 Btaf1 Rpl14 Dok2 Sh2d2a Aip Pstpip1 Nfs1 Prc1 Slc7a6 Papss1 Pias2 Syt7 Cldn12 Ash2l Cldn2 Cldn9 Pkmyt1 Pigq Sart1 Dnaja3 Unc119 Tbx19 Usp14 Usp2 Usp10 Usp8 Mtmr6 Mtmr4 Nmi Mta1 Lats1 Atp6v0d1 Sec22c Slc16a6 Slc16a5 Slc16a3 Pdlim1 Smc3 Prpf3 Aifm1 Ccnb2 Ccne2 Arhgef1 Cbfa2t2 Atg12 Pdcd5 Syngr1 Nemf Dyrk1b Ctdp1 Slc28a2 Gm14085 Slc28a1 Exo1 Fibp Pcsk7 Dgki Ebag9 Cox7a2l Tmsb10 Gm9844 Scaf11 Myom2 Arhgef2 Zw10 Bub3 Ddx21 Dedd Slc16a7 Kcnab3 Slc33a1 Zmym4 Zmym6 Zmym5 Lrrfip1 Lrrfip2 Lgi1 Aurkb Vapb Vapa Mta2 Nolc1 Rab11b Dlg5 Pttg1 Ccpg1 Tbrg4 Pnma1 Dhrs3 Rps6ka5 Aimp1 Mfhas1 Mapkapk2 Cyth2 Cyth1 Itgb1bp1 Bcl7c Bcl7b Copb2 Zbtb22 Med14 Gpr55 Srsf11 Atp6v1f Socs6 Cd83 Zfp235 Pter Cops2 Trip12 Trip11 Hmgn3 Trip4 Znhit3 Gtf3c5 Gtf3c3 Cnot8 Tceal1 Vamp3 Eftud2 Taok2 Rpl23 Slc9a3r2 Txnl1 Ube4a Itgbl1 Ppig Lonp1 Rab33a Rab28 Slc9a3r1 Adipoq Kif3b Zfyve9 Plaa Ppt2 Tm9sf2 Cox5a Nrxn2 Grhpr Otof Cog1 Ciao1 Hs6st1 Nmt2 Cd101 Stoml1 Grap2 Lpxn Zranb2 Pex16 Med21 Fam189a2 Fads2 Ddx23 Cript Med23 Med17 Med26 Med27 Med7 Itm2b Gsto1 Map4k4 Ly86 Eif2ak3 Itm2a Ggps1 Homer1 Fhl5 Arhgef6 Pick1 Akap7 Il27ra Sh3bp5 Eif4e2 Akap6 Atg5 Rock2 Napsa Med20 Gm20517 Slc25a27 Stx8 Chst10 Pigl Pigb Pgs1 Psmf1 Kif23 Akap5 Slc4a7 Myot Maged1 Rph3al Pmpcb Fxr2 Litaf Sptlc2 Tbpl1 Npepps Scamp1 Tecr Vps4b Mpdu1 Gosr1 Tmem59 Bag3 Bag2 Gmfg Ptges Ei24 Cir1 Nrg2 Cxcl14 Atp6v1g1 Atp5j2 Spag7 Mrpl33 Sec22b H2afy 2010107E04Rik Chd1l Vps26a Minpp1 H6pd Gtpbp1 Gosr2 Nr1d1 Clock Spag6l Spag6 Entpd4

			Gm21685 Rbm39 Kif20b 9430076C15Rik Mad2l1bp Prdx6 Prdx6b Wtap Ier2 Cytip Pitpnm1 Pdia4 Nfe2l3 Rnf14 Cartpt Ncor1 Ncor2 Gm7075 Rnf7 Mtrf1 Traf4 Abcg1 Nup155 Sec24c Isg15 Zfp592 Ikbke Morf4l2 Sh3pxd2a Mical2 Ctr9 Gcc2 Ralgps1 Hs2st1 Ttll4 Socs5 Mdc1 Iqcb1 Zfp516 Pde4dip Cep135 Lpin2 Marf1 Safb2 Eif5b Ipo13 Sdc3 Slc25a44 Phf14 Fam53b Depdc5 Kdm4a N4bp1 Lrrc14 Clint1 Vgll4 Nup93 Bzw1 1110008L16Rik Rapgef2 Crocc Tram2 Pum1 Rims2 Espl1 2610507B11Rik Dhx34 Ulk2 Herpud1 4931406P16Rik Usp6nl Aqr Tmem63a Zfp646 Secisbp2l Sart3 Kntc1 Usp34 Ccp110 RP23-325D10.3 Setd1a Laptm4a Ift140 Arhgap32 Acap1 Clstn3 Slk Fam65b Snph Tbkbp1 Hdac4 Tox Mlec 6430548M08Rik Zfyve16 Rassf2 Bclaf1 Eif4a3 Atg13 Tm9sf4 D5Ertd579e Tbc1d5 Rnf144a Matr3 Dhx38 Dlgap5 Mtss1 Bms1 Ptdss1 Sertad2 Ckap5 Maml1 Phyhip Tatdn2 Ist1 Mrpl19 Dazap2 Tomm20 Spock2 Ip6k1 Rnf40 Ctif 0610009O20Rik Sfi1 Git2 Urb2 Keap1 Tsc22d2 Cul7 Rb1cc1 Arhgap11a Spata2 Trim14 Zfp623 Melk Lcmt2 Gins1 Zbtb24 Elmo1 Mfap3l Zfp518a C2cd2l Farp2 D130043K22Rik Cep350 Lrig2 Psmd6 Med24 Pja2 Setdb1 Sec24d Fchsd2 Tlk1 Urb1 Zc3h11a Tox4 Ddx46 Zbtb39 Trank1 Tbc1d4 Pom121 Lrrc37a Gm884 Osbpl2 Smg7 Telo2 E430025E21Rik Sv2b Srgap3 Mrc2 Klhl21 Rbm19 G3bp2 Dennd4b Tmcc2 Supt7l Atp2c2 Fam20b Sec16a Kbtbd11 Rnf10 Iqsec1 Zbtb40 Pan2 Zbtb5 Lpgat1 Mfn2 Kif14 Josd1 Helz Arhgap25 Rbm8a Rbm8a2 Dlec1 Exog Xylb Oxsr1 Gfpt2 Cryzl1 Wdr1 Golga5 Hs3st3b1 Hs3st3a1 Hs3st1 Usp15 Usp3 Mvp Slc23a2 Fgf15 Thrap3 Med13 Nup153 Nr1d2 Dopey2 Thoc1 Rce1 Dmtf1 4932411N23Rik Gm21985 Slc12a6 Dgcr2 Gm20518 Casp8ap2 Med6 Acot8 Abi1 Gnpda1 Kcne3 Zbtb33 Tank Hdac6 Hdac5 Pdcd6ip Pdcd6 Bcl2l11 Gne Peg12 Frat1 Troap Med16 Pigk Chaf1a Parp2 Parp3 Hmgxb4 Tom1 Sh2d3c Ranbp9 Dnajb6 Smc4 Ap1m2 Uba2 Sae1 Farsb Abcc5 Dnm1l Abcf2 Gm21987 Cox17 Scamp2 Scamp3 Il18bp Rwdd2b Dpp3 Snupn Huwe1 Tssc4 Pdcd7 Pqbp1 Ust Gm16372 Arpc3 Arpc1b Actr3 Actr2 Tspan5 Tspan3 Tspan2 Nubp2 Tsfm Ppif Ctdsp2 Arpc2 Rad50 Kif20a Preb Hipk3 Fem1b Actr1b Actr1a Arl4c Arl4a Rasgrp1 Dnal4 Zfp263 Lrpprc Pdia6 Trap1 Bcap31 Nampt Yaf2 Arfrp1 Tob1 Akap9 G3bp1 Sugp2 Mbnl2 Abi2 Cebpz Plxnc1 Trim28 Pdzk1ip1 Atp6ap2 Lpcat3 Wasf2 Slc25a13 Slc25a15 Serf2 Dhrs9 Rcl1 Rbm7 Rbm6 Rbm5 Tnk2 Txndc9 Rnf41 Tshz1 Alg3 Prmt3 Psme3 Mphosph9 Mphosph10 Mphosph6 Nme6 Nutf2-ps1 Nutf2 Uspl1 Eif1 Topors Ddx39 Psmd14 Olig2 Ctdspl Gdf11 Trib1 Gpa33 Stx6 Coq7 Lrrc23 Rasgrp2 Hnrnpr Slc35b1 Dcaf7 Ap3s2 Mrps31 Calcoco2 Gphn Rabepk Pop7 Srrm1 Spry1 Spry2 Stam2 Abcc4 Dennd4a Igsf6 Sf3b4 Cdk2ap2 Irx5 Ramp1 Ramp3 Zmpste24 Akap8 Stub1 Stag1 Net1 Ube4b Prss16 Sigmar1 Bet1 Sap18b Sap18 Smndc1 Bcas2 Rgs19 Eif1b Sf3a1 Traip Dnaja2 Bckdk Maea Apc2 Pak4 March6 Katnb1 Snapc5 Sra1 Dscr3 Rtn3 Lancl1 Nmur1 Tnip1 Ikzf1 Smyd5 Rragb Akr1a1 Tmem5 Cnpy2 B3gnt3 Tfg Abca7 Wars2 Npm3 Npm2 Hmg20b Hmg20a Micu1 Cited2 Sema3a Tuba1b Bpnt1 Tubb3 Tubb4b Nod1 Anapc10 Atp8a1 Ndrg1 Pemt Yap1 Spon1 Prmt5 Tesk2 Cd2bp2 Ubac1 Pgrmc2 Arih2 Tubgcp3 Sec24b Cfdp1 Tmem147 Rbm14 Emg1 C1d Timm17a Zer1 Mcrs1 Lrrn2 Fam3c Acaa2 Ppie Vav3 Tomm40 Tab1 Eci3 Eci2 Hax1 Baiap2 Mad2l2 Tacc3 Slc30a9 Pibf1 Gm7879 Timm44 Pfdn6 Tada3 Gm10250 Atp5h Ube2e3 Slc9a6 Eif3m Nxf1 Sec23b Sec23a Cap1 Creb3 Lrrc41 Vti1b Crtap Syncrip Vat1 Stk25 Enox2 Carm1 Ncoa2 Sema4f Sema4d Appbp2 Mybbp1a Cib2 Cib1 Ddx17 Deaf1 Cherp Kat5 Hyou1 Ipo8 Ipo7 Nop56 Pitrm1 Atg7 Sssca1 Rnaseh2a Batf Glrx3 Dctn2 Anp32b Tm9sf1 Arl6ip5 Arpc1a Agpat1 Agpat2 Rpp30 Sptlc1 Slc35a1 Arfgef2 Arfgef1 Rabac1 Slu7 Siva1 Mrpl28 Cct7 Cct4 Cct2 Pomt1 Txnrd2 Mthfs Mthfsl Drap1 Smc2 Prpf8 Usp16 Cdc42ep3 Paip1 Paics Tbl3 Mxd4 Erlin1 Hexim1 Spag5 Rbck1 Stambp

			Tgoln1 Arid3b Polr3f Ivns1abp Trim16 Txnip Taf6l Gm5426 Atp5l Rgs14 Lefty1 Lefty2 Nprl2 Igf2bp3 Camkk2 Mtx2 Ykt6 Pmvk Khdrbs1 Celf1 Celf2 Cxcr6 Ctcf Cd226 Fars2 Cgrrf1 Rraga Dctn6 B3gnt2 Gnb5 Ebp Gmeb1 Cct6b Cct8 Cnpy3 Fam189b Usp39 Pold3 Ap4b1 Gm43064 Polq Slc12a7 Mgea5 Nfat5 Nudc Ptges3 Plk4 Stag3 Six2 Rbbp9 Rai1 Phtf1 Kif1c Grap Gipc1 Traf3ip2 Nes Kdm5b Hbs1l Ahcyl1 Zmynd11 Srsf10 Zbtb6 Pop4 Zfp369 Zfp110 Nek6 Wdr4 Iqgap2 Mthfd2 Rpp40 Cysltr1 Sept9 Sec24a Ccr9 Sdccag8 Hsph1 Stard10 Utp14b Utp14a Cplx2 Cplx1 Frs3 Frs2 Neu3 Clpx Gm42715 Srcap Ppp1r13l Ccl27b Ccl27a Gm13306 Ruvbl2 Cyp46a1 Arid5a Usp20 Usp19 Hcst Mrps30 Rab10 Ppargc1a Yif1a Jtb Blcap Man1a2 Trafd1 Txnl4a Pnpla6 Sugt1 Gadd45g Edar Papola Tcerg1 Ehmt2 Cops8 Rnps1 Fastk Sub1 Spin1 Ralbp1 Morf4l1 Prdx3 Ehd1 Afg3l2 Msl3l2 1110004F10Rik Kdelr1 Sf3a3 Ap3m2 Stard3 Hnrnpa0 Gm7334 Btg3 Cbx1 Gm10320 Sec61b Tomm34 Pdia5 Serinc3 Os9 Pnrc1 Tmed2 Lman2 Mllt11 Stip1 Ywhaq Tmed10 Ascc3 Uqcr11 Clp1 Fermt2 Cops6 Mapre2 Ccni Cops5 Metap2 Immt Sf3b2 Ilvbl Slc27a5 Slc27a4 Slc27a3 Kif2c Ccdc85b Kdelr2 Atf7 Gm28047 Snrnp27 Tmed1 Ift27 Rab35 Tdrkh Adap1 Dstn Pim2 Papd7 Gm9774 Adrm1 Cpsf6 Ogfr Zpbp Ddx52 Abhd2 Wwp2 Dus4l Cntrl Ube2c Snrnp35 Tmem115 Topbp1 Trim31 Tppp Hsf2bp Rer1 Dnajb4 Dido1 Wdr5 Nupl2 Hnrnpul1 Ate1 Rpp14 Krr1 Katna1 Prdm4 Hibadh Cit Fgfr1op Emilin1 Btn2a2 Faf1 Kif3a Polr3a Clasrp Zwint Capn10 Kptn Slc7a9 Pwp1 Tbc1d8 Cdc37 Pkig Kat7 Glmn Coro1a Wdr45 Ficd Ap4s1 Ldb3 Ptp4a3 Lsm6 Rabl2 Erlin2 Nudt4 Nudt3 Fstl1 Psip1 Strap Adamts7 Baz2a Baz1a Lzts1 Zfp277 Wdr6 Treh Slc2a6 Map4k5 Map4k1 Inmt Rassf1 Nisch Cep250 Wbp4 Abcb8 Sec23ip Supt16 Chek2 Poli Prosc Akap13 Akap11 Akap10 Akap2 Pakap Dusp10 Mrpl3 Gm10269 Gm2000 Rpl35 Galnt6 Praf2 Sec63 Polg2 Pdcd10 Rnf139 Rnf24 Pmf1 Zhx1 Nxph3 Man1b1 Dctn3 Xpot Pxmp4 Dusp12 Snf8 Nrm Atxn2l Usp18 Synrg Gm42641 Trex1 Klf12 Pnkp B4galt7 Slco2b1 Vps45 Lypla2 Cd300c Cd300c2 Cd300a Park7 Cope Rbpjl Ecd Mgat4a Gpn1 Tmc6 Vsig4 Acot7 Tusc2 Gabarap U2af2 Oip5 Exosc8 Rnf13 Twf2 Gabarapl2 Casc3 Cog2 Rras2 Xrn2 Ikzf2 Copz1 Rasa3 Phlda1 Mtf2 Dnajc8 Puf60 Scaf8 Zfp652 Zfp30 Rhobtb3 Rnf44 Dlgap4 Rab11fip2 Vash1 Zfp507 Aak1 Cpeb3 Adnp2 Ankrd26 Lmtk2 Chsy1 Ick Fndc3a Atg14 Fastkd2 Ppp6r1 Nlgn1 Sec31a Plekha6 Enpp4 Inpp5f Trappc8 Mon1b Zhx2 Wdr37 Foxj3 2810403A07Rik Zbtb1 Zfp365 Cep164 Dennd3 Arsg Rufy3 Btbd3 Sbno2 Dhx30 Sacm1l Fan1 Wdr47 Raly Klrk1 Mmrn1 Ncbp2 Mapre1 Kifap3 Atf6 Sephs2 Sephs1 Rab3gap1 Rab18 Sirt2 Rpia Scap Snw1 Cct5 P2rx2 Tpx2 Tcf25 Ninl Dip2c Pdcd11 Sorcs3 Kdm2a Cep152 Ttc39a Igsf9b Daam1 Mapkbp1 Klhdc10 Rab21 Stk38l Spen Fbxo21 Exosc7 Cnot1 Snrnp200 Palld Tmcc1 Kdm1a Rbm34 Kdm4b Mast3 Usp33 Dopey1 Zfp292 Wdtc1 Xpo7 Myt1l Mon2 Pdxdc1 Tnik Kif21b Pds5b Fnbp1 Smg1 Zhx3 Cluap1 Gm20695 Zfp609 Gga2 Setx Setd1b Erp44 Swap70 Rrp1b Mycbp2 Avl9 Kdm4c Pprc1 Erc1 Zc3h13 Arhgap26 Ttll5 Sipa1l3 Kif1b Cdk19 Zbtb43 Tbc1d2b Mrps27 Rap1gap2 Tnrc6b Nfasc Fam179b Tab2 Clasp2 Camta2 Pogz Plxnd1 Atg2a Gpatch8 Rad54l2 4921501E09Rik Phf8 Kdm6b Smc5 Mast2 Ankle2 Dcun1d4 Lrch1 Zc3h3 Fcho1 Cic Ncdn Clcc1 Wdr43 Snx13 Mapk8ip3 Mprip Nup205 Efr3a Rtf1 Slc35d1 Gpd1l Fam175b Metap1 Zcchc14 Lpin1 Sept8 Cep68 Rftn1 Dip2a Mesdc2 Larp4b Rcor1 Kank1 Ubxn4 Atg4b Ganab Mdn1 Fam120a Faf2 Psme4 Atp11b Fam168a Pmpca Arl6ip1 Syt11 Jmjd6 Zc3h4 Rrs1 Xpo6 Prrc2c Nbeal2 Fbxo28 Dtx4 Rhobtb2 Rrp12 Nup210 Plcl2 Arhgef9 Vps13a Exoc6b Dnajc9 Sik2 Plcb1 Arc Phlpp1 D930015E06Rik Pacs2 Cobl Ankrd28 Pds5a Astn2 D430042O09Rik Rprd2 Atp11a Otud3 Ankrd12 Kazn Scfd1 Dennd5a Ddhd2 Camta1 Mcf2l Exoc7 Mga Tspyl4 Camsap2 Fam208a Clec16a Pofut2 Klhl18 Nup160 Cstf2t Wwc1 Agtpbp1

			Iqce Fbxw11 Smg6 Anks1 Mgrn1 Bicd2 Atmin Ehbp1 Kif13b Ubr2 Acsl6 Fkbp15 Icosl Sin3b Ncapd3 Dmxl2 Satb2 Slc9a8 Cux2 Dnajc13 Trim2 Man2b2 A230046K03Rik Usp22 Sash1 Clasp1 Wdr7 Synm Vps39 Dnajc16 Esyt1 Smchd1 Dock9 U2surp Khnyn Ubr4 Sun1 Haus5 Vps8 Angel1 Usp24 Fnbp4 Arhgef12 9530077C05Rik Larp1 Ppp1r13b Pum2 Arhgef18 Rrp8 Ahcyl2 Mau2 Specc1l Ncstn Nudcd3 Med13l Zdhhc17 AI314180 Adnp Lars2 Ncaph Ppwd1 Atp13a2 Frat2 Fbxo46 Exosc2 Cotl1 Sirt5 Sirt4 Sirt1 Commd3 Ncs1 Mlycd Crb1 Tmed3 Tdrd7 Rybp Ap4e1 Rhoq Tardbp Hars2 Slc35a3 Slc44a1 Sf3b3 Sf3b1 Abcb10 Hey1 Icmt Gcat Cbx5 Phf3 Tram1 Capn7 Ethe1 Qprt Sec11a Iscu Sec61g Pes1 Tgds Leprotl1 Ces3a Ces3b Cbx7 Hey2 Haao Macf1 Zfyve26 Tmem131 Ttc9 Pofut1 Kctd2 Nup188 Suz12 Scrib Morc3 Skiv2l2 Rpl13a-ps1 Rpl13a Kat6b Cabin1 Srrm2 Acap2 Zfp281 Nnt Pik3r5 Tnpo3 Adat1 Rbfox2 Atp6v0a2 Ttc33 Dnpep Psd4 Rasd2 Cdk20 Tspan15 Pign Snapin Wbp2 Wbp1 Gtpbp4 Cldn14 Lpar3 Zfp346 Arl2bp Cdc42ep4 Ccndbp1 Smug1 Tmem50a Ddx58 Klhdc2 Carhsp1 Pdss1 Lemd3 Hebp2 Orc6 Patz1 Amacr Coro1c Dapk2 Cd2ap Mkrn1 Mkrn2 Phlda3 Zmynd8 Sh3bp1 Rusc1 Kpna6 Ssbp2 Nup62 Rabgap1 Lrrc6 Hspbp1 Ly96 Edc4 Ppp1r15a Pld3 Ssbp3 Pola2 Lsm5 Pla2g15 Zkscan5 Tmem2 Stx12 Prkd3 Gspt2 Gabarapl1 Eid1 Aipl1 Sdf2l1 Ppil2 Pitpnb AC149090.1 Il17ra Fkbp8 Bcl2l13 Mtch1 Mtch2 Kif4 Eml2 Ftsj1 Nat6 Tfip11 Panx1 Prpf6 Zfp318 Prpf40b Cby1 Sun2 Dstyk Rab3gap2 Rad54b Ciz1 Fbxo7 Pgls Qpct Zfp324 Lsm4 Tnfaip8 Arih1 Mto1 Dnajb5 Tpsg1 Txn2 Tmem184b Hectd1 Nipbl Abtb2 Asf1a Mob4 Yipf3 Tecpr1 Armc8 Dcaf12 Brms1 Usp49 Abhd14a Prkd2 Sumf2 Gm8973 Letmd1 Spef1 Polr1a Poc1a Zfp473 Plekhg4 Rnf19a Iffo1 Ccdc28a Mthfd1l Cnot10 Ahctf1 4933434E20Rik Pot1b Pot1a Rttn Ndufaf3 Thumpd3 Zdhhc5 Atl3 Nol11 Gemin5 Ptpn23 Nipsnap3a Nipsnap3b Heatr5a Samhd1 Fam98a Sin3a Kbtbd2 Syf2 Rwdd3 Pnkd Sec31b Pnisr Nudt13 1110037F02Rik Tmem87a C2cd2 Sh2b1 Unc50 Pars2 Mmachc Tiparp Necap1 Chmp2b Dhrs7b Ngdn Tsku Hinfp Ulk3 Higd1a Rexo2 Ibtk Tbc1d10b Rnf167 Gorasp2 C2cd3 Zzz3 Rpap1 Fam32a Lrig1 Upf2 Lrp10 Ptcd1 Acot11 Plekhg3 Osbpl3 Ipcef1 Glce Zfp451 Setbp1 Ubxn7 Lrrtm2 Ltn1 Fam169a Ppp1r16b Auts2 Rab11fip5 Ankrd17 Gigyf2 Appl1 Hacl1 Decr2 Rai14 Lsm14a Cxx1c Cxx1a Cxx1b Poldip2 Gga1 Abhd12 Herc4 Tor1aip1 Ccdc9 Dcaf4 Chtop Wipi2 Ccdc69 Wsb1 Prpf31 Epc2 Tctn3 Gapvd1 Trpc4ap Serbp1 Zbtb20 Irf2bp1 Traf3ip1 Phf19 Nat9 Kif26a Noc2l Rsl1d1 Ift172 Senp3 Ints1 Fbxw2 Ptpn22 Gmeb2 Spag8 Pitpnc1 Fbxl3 Arl5a Stap1 B3gat3 Tiam2 Fbxl5 Fbxl4 Optc Fbxw8 Fbxo25 Fbxo24 Tspan17 Fbxo22 Fbxo9 Fbxo5 Fbxo4 Fbxo3 Hibch Vps33b Tinf2 Sacs Eral1 Mycbp Sergef Ehf Gbgt1 Gnl3 Ptpn18 Ppp1r14b 4933415F23Rik Narf Slc17a5 Heyl Myof Chic2 Ints6 Timm13 Timm10 Timm9 Lats2 Dazap1 Aatf Ostf1 Bscl2 Ckap2 Mrpl46 Tbl2 Elp4 Olfr1509 Olfr464 Nufip1 Rps6kc1 Sh3yl1 Gm26387 Gm26109 Gm22200 Rnu11 Ranbp6 Copg2 Hbp1 Chordc1 Sec22a Ap3m1 Pabpc2 Akap8l Rnf11 Gpr160 Cyfip2 Dnajc2 Usp21 Tpk1 Cnppd1 Nptn Foxb1 Atp2c1 Trmt2a Pkd2l2 Lat Diexf Pelp1 Snd1 Ankrd1 Stau2 Ppa2 Ghitm Vps41 Tspan13 Lypd3 B9d1 Rpusd2 Uqcrq Trappc3 Cacybp Eif2ak1 Arrdc2 Zbtb11 Atp5s Sdcbp2 Pde7b Aff4 Cyth4 Snx5 Cpne7 Tjp3 Stk36 Zfp777 Brpf3 Naaa Sall3 Slc39a1 Tubg2 Il1f6 Siglece Vps4a Tubgcp4 Serp1 Coq2 Gpkow Bbs9 Tnfrsf21 Chmp2a Sesn1 Ahdc1 Rnf115 Nfu1 Mmadhc Pdcd4 Klhl20 Lsm1 Lsm3 Sult1b1 Dimt1 RP23-218K15.5 Dhdh Crcp Apex2 Bmp10 Rbms3 Zfp330 Rab30 Tnrc6a Zfp638 Eif3k Htatsf1 Ube2s Prpf19 Utp20 Rabgef1 Tmem97 Stk39 Tor1b Sgsm3 Mat2b Tor2a Polm Eml4 Cecr6 Cecr5 Slco3a1 Nkiras2 Nkiras1 Dll1 Trib2 Ccdc22 Dexi Lamtor2 Mrps28 Dcps Ostm1 Git1 Bzw2 Mrps18b D8Ertd738e Acad9 Mrpl42 Tmem14a Mfsd7b Mcts1 Mageh1 Nob1 Dbnl Commd5 Macrod1 Hipk2 Mrpl13

			Slc43a3 Atad2 1810013L24Rik Tmem29 Zcchc4 Zc3h7a Ccdc113 C1galt1c1 Setd2 Ndufaf4 Med4 Ccdc59 Mettl5 Gtpbp8 Phpt1 Babam1 Thyn1 Mrpl15 Ube2t Mrpl22 Ormdl2 Cnih4 Rangrf Commd9 Tmem208 Ssu72 Drosha Dnajc15 N6amt1 Nxt1 Tbk1 Sap30bp Mylip Brd7 Ctnna3 Ankrd11 Racgap1 Usp25 Tmod3 Tmod2 Abt1 Ola1 Ypel1 Repin1 Senp1 Icos Alg5 Cnot7 Snx10 Gnl2 Rbm15b Psmc3ip Cpsf1 Mylpf Tra2a Gpsm2 Sac3d1 Ccdc106 Eef2k St8sia5 Snx15 Hook2 Ubiad1 Hcfc2 Snx11 3110002H16Rik Pycr2 Epn1 Gmppb Gmppa Sec61a1 Timm22 Alg6 Gpr132 Snx12 Nenf Purg Padi1 Pnma3 Anapc4 Sertad3 Il19 Sertad1 Dpp7 Cers2 Slc25a24 Strn3 Lrp12 Psat1 Ubqln1 Donson Nrbf2 Slc39a3 Slc2a8 Pilra Pilrb1 Pilrb2 Lmcd1 Gltscr2 Gltscr1 Tnpo2 Efemp2 Tbx21 Sh3kbp1 Slc40a1 Hunk Vsx1 St6galnac6 Kcnip2 Kcnip1 Cxxc1 Nt5c Znrd1 Dnttip2 Socs7 Ehd4 Ehd3 Pik3r4 Cdr2l Stoml2 Scaper Wrap73 Crnn Rrm2b Smarcal1 Mink1 Chst11 Galnt9 Il22 Iltifb Atp6v0a4 Itsn2 Def6 Cyhr1 Gemin4 Pnpla8 Arhgef4 Arhgef3 Myef2 Gm9833 Irx4 Asap1 Ak3 Hp1bp3 Cops7a Nsdhl Dhh F11r Vill Stmn3 Rnf141 Cdon Sost Tmed5 Slc35b3 Tprkb Med31 Coq6 Amdhd2 Slc35c2 Exosc3 Fahd2a Exosc1 Isoc1 Rrp15 Ccdc53 Hddc2 Mrps16 Mrps18c Fis1 Bola1 Vps36 Glod4 Ubxn1 Zfp593 Zbtb7b Gmnn Lap3 Zfp691 Txndc12 Txndc11 Rps27l Yars2 Nmd3 Mrpl2 Nosip Dera Memo1 Mrpl4 Apip Cutc Thap4 Ndufa13 Mrps7 Polr1d Gal Cryl1 Tnni3k Pllp Sepsecs Sidt2 Rrnad1 Adipor1 Trnt1 Utp18 Sccpdh Ift52 Abhd5 Sh3glb1 Mecr Ndufaf1 Phf20l1 Tfb1m Aph1a Mettl9 Rdh11 Mrps2 Coq4 Sbds Commd2 Zfp706 Gm10784 Ier3ip1 Golga7 Sar1b Asb3 Gm6904 Phf11a Phf11b Phf11d Phf11c Rlim Kctd3 Rnft1 Cops4 Insig2 Chchd2 Dync1li1 Hsd17b12 Ing4 Sdf4 Mrto4 Hn1 Zfp580 Dbr1 Dctn4 Cyb5r4 Hsd17b11 Hsd17b14 Nagpa Tubd1 Lef1 Plekho1 Hspa14 Gpn3 Crbn Rsl24d1 Deb1 Cklf Ipo11 Nin Zdhhc2 Ddx47 Taco1 Acp6 Gp6 Zfp219 Abi3 Gltp Phf20 Vrk3 Crim1 Mzb1 Ankrd39 Cox16 Shisa5 Paip2 1700021F05Rik Fam178b Mrpl37 Rnf181 Tbc1d7 Mrpl51 Mrpl30 Mrpl27 Cdkl3 Ubap1 Bet1l RP23-114A6.7 Dnajc27 Ier5 Scand1 Bfar Ergic2 Gmip Gmpr2 Cd320 Pcdh12 Ecsit Nrn1 Timmdc1 Gcnt4 Fkbp11 Fam13b Fam53c Slc25a37 Krcc1 Plac8 Phf21a Mrpl35 Rsrc1 Mex3c Amz2 Wac Spg21 Arl6ip4 Zfp771 Ngrn Zbtb7a Fzr1 Taok3 Mbtps2 Hook

Supplementary Table 3. Analysis of upregulated regulons in stressed *Tph2*$^{+/-}$ vs. wildtype female mice. Acronyms of regulons (columns **A**) refer to gene names of corresponding transcription factors. Involvement of known transcription factors in the genome-wide transcriptional responses to stress on wild type (column **B**) and mutant (column **C**) backgrounds. Gene targets of the transcription factors are listed in a column **D**. Worksheet 1: stimulated regulons, worksheet B: suppressed regulons. Numbers in the columns B and C are -log10(FDR), where FDR is the false discovery rate associated with the involvement of the respective regulon in the transcriptional response. FDR=0.05 corresponds to the table value of approximately 1.3.

Supplementary Table 4

Regulons	Stress, Tph2+/+ background	Stress, Tph2+/− background	Target's List
Sox17	0,71	3,22	Abi3bp Acvrl1 Adamts9 Afap1l1 Alpk2 Angpt2 Anxa3 Arap3 Arhgap29 Arhgdib Arnt2 Arrb1 Arsj Art4 Atf6 B4galt6 Bicc1 Bmp2 Bmp4 Bmp6 Bmpr1a Bmx 8430408G22Rik 5730508B09Rik Calcrl Card6 Casp12 Cd93 Cdh5 Cdk17 Cep112 Chst1 Cldn5 Clec14a Clec1a Cmtm7 Cntnap3 Col18a1 Col8a1 Copz2 Crip2 Crispld2 Csf2rb Csf2rb2 Cxcr4 Cxxc5 Cyyr1 Daam2 Dach1 Dchs1 Dcn Dhh Dnm1 Dock4 Dock6 Dock9 Ecscr Edn1 Efemp1 Egfl7 Ehd4 Elmo1 Emcn Entpd1 Eogt Epb41l4a Ephx4 Erg Esam Esm1 Fabp5 Fam124b Fam198b Fam43a Fam49a Fam69a Fam69b Far2 Fblim1 Fbxl13 Fermt3 Fgd5 Fli1 Flt1 Flt4 Foxc2 Frmd4a Frmd4b Fry Fstl5 Fzd4 Fzd7 Gbgt1 Gfod1 Gfra1 Gimap1 Gimap8 Gja4 Gng11 Gpc4 Gpc6 Gpr161 Gpr4 Grap Hcls1 Hddc2 Hecw2 Hhex Hhip Hoxd1 Hspa12a Hspa12b Hspg2 Hyal2 Ica1 Icam2 Ifi27 Igfbp5 Il18r1 Inpp5d Irf6 Irs1 Itga2 Itm2c Itpkb Jag2 Jam3 Kank3 Kcnma1 Kctd12 Kdr 2810474O19Rik Kif26a Klhl13 Klhl4 Klhl6 Lama4 Laptm5 Lims1 Lrrc8c Lum Lyl1 Maml3 Mansc1 Mast4 Mcam Mctp1 Mecom Meox2 Mertk Mfng Mgat4a Mgp Mipol1 Mmp10 Mmrn1 Mmrn2 Mpp4 Mpzl2 Mrpl22 Myct1 Myrip N4bp3 Neo1 Nos3 Notch1 Nr5a2 Nrarp Nrg3 Nsun5 Olfml2b Palmd Pcdh12 Pcdh17 Pde10a Pde3a Pdgfb Pdgfra Pdgfrb Pear1 Pecam1 Peli1 Phldb2 Pik3r3 Pir Pld1 Plekhg1 Plk2 Plxna2 Prex1 Prex2 Prkacb Prkar2b Prkch Procr Ptger4 Ptprb Ptpre Rarb Rasip1 Rassf9 Rbms2 Rbpj Rgs3 Rgs5 Rhoj Rnase1 Robo4 Runx2 S1pr1 Samsn1 Scarf1 Sdpr Sec14l1 Sema3c Sema3f Serpinb9d Serpinb9e Serpinb9f Gm11397 Serpinb9g Serpinb9 Serpinb9b Serpinb9c Sh2d3c Sh3tc1 Shank3 She Shroom4 Snap23 Snca Snrk Sntb1 Sox18 Sox7 Spon2 Srgn Srpx2 St6gal1 St6galnac3 St8sia4 Stab1 Stx11 Styk1 Sult1b1 Susd5 Sytl2 Tacc1 Tal1 Tanc1 Tctex1d1 Tead3 Tek Tfpi Tfpi2 Tgfbr2 Thsd1 Thsd7a Tie1 Tjp2 Tlr4 Tm4sf1 Tnfrsf10b Tnfrsf11a Tpk1 Trps1 Usp31 Utrn Uvrag Vash1 Vdr Veph1 Vip Vwf Wwtr1 Yap1 Zhx3 Zfp422 Zfp239 Zfp521
Sox18	0,25	3,22	Ace Acvrl1 Adam15 Adam9 Adamts9 Afap1l1 Alpk2 Angpt2 Ano2 Anxa3 Apln Arap3 Arhgap29 Arhgdib Arhgef15 Arhgef7 Arnt2 Arrb1 Art4 B4galt6 Bcl6b Bicc1 Bmp4 Bmp6 Bmpr1a Bmpr2 Bmx 8430408G22Rik C1qtnf6 5730508B09Rik Calcrl Card6 Casp12 Cd151 Cd34 Cd55b Cd55 Cd59b Cd59a Cd93 1700027J19Rik Cdh13 Cdh5 Cdk17 Chst1 Cldn5 Clec14a Clec1a Col13a1 Col8a1 Copz2 Crip2 Crispld2 Csf2rb Csf2rb2 Cxcr4 4930578C19Rik Cxxc5 Cyb5r3 Cyp1a1 Cyyr1 Dach1 Dchs1 Dcn Dhh Dnajb4 Dnm1 Dock4 Dock6 Dock9 Dusp18 Dysf Ece1 Ecscr Edn1 Efemp1 Efna1 Egfl7 Ehd2 Ehd4 Eif2b2 Elk3 Elmo1 Emb Emcn Eng Enpp2 Entpd1 Eogt Ephx4 Erg Esam Esm1 F2r Fabp4 Fabp5 Fam124b Fam198b Fam43a Fam49a Fam69a Fam69b Far2 Fblim1 Fermt3 Fgd5 Fkbp1a Fli1 Flt1 Flt4 Foxc2 Frmd4a Fry Fzd4 Fzd7 Gbgt1 Gfod1 Gfra1 Gimap1 Gimap4 Gimap6 Gimap8 Gja4 Gng11 Gpc4 Gpc6 Gpr161 Gpr4 Hcls1 Hecw2 Hhex Hhip Homer3 Hoxd4 Hspa12a Hspa12b Hspg2 Hunk Hyal2 Ica1 Icam2 Ifi27 Inpp5d Irs1 Itga2 Itga5 Itm2c Itpkb Jag2 Jam3 Kank3 Kdr Kif26a Klf5 Klhl4 Klhl6 Klrg1 Lama4 Laptm5 Lgals1 Lrrc32 Lrrc8c Lum Lyl1 Mall Maml3 Mapre2 Mcam Mctp1 Mecom Meox2 Mertk Mfng Mgp Mipol1 Mmp10 Mmrn1 Mmrn2 Mpp4 Msrb3 Myct1 Neo1 Nlrp1a Nlrp1b Nod1 Nos3 Notch4 Nrg3 Nrgn Nrp1 Pald1 Palmd Parp8 Pcdh12 Pde3a Pde4b Pdgfb Pdgfra Pdgfrb Pear1 Pecam1 Pik3r3 Pir Piwil4 Pkn3 Pld1 Plekhg1 Plk2 Plod1 Plxna2 Pomgnt1 Ppm1f Prex1 Prex2 Prkar2b Prkch Procr Prss23 Ptger4 Ptprb Ptpre Ptrf Ramp2 Rarres2 Rasip1 Rbms2

			Rbpj Rgs3 Rgs5 Rhob Rhoj Rnase1 Robo4 Rps6ka2 Rras Runx2 S100a16 S1pr1 Samsn1 Sdpr Sec14l1 Sema3f Sema6b Serpinb9d Serpinb9e Serpinb9f Gm11397 Serpinb9g Serpinb9 Serpinb9b Serpinb9c Sh2b3 Sh2d3c Sh3tc1 Shank3 She Shroom4 Sipa1 Slc29a1 Smtn Snap23 Snca Sntb1 Sox17 Sox7 Srgn Srpx2 St6gal1 St6galnac3 St8sia4 Stab1 Stx11 Styk1 Sytl2 Tal1 Tax1bp3 Tecpr1 Tek Tfpi Tgfbr2 Thbs1 Thsd1 Tie1 Tm4sf1 Tmem88 Tnfaip1 Tnfrsf10b Tnfrsf11a Tnfrsf25 Tnfsf18 Triobp Trps1 Tspan15 Tspan9 Txnrd2 Usp31 Utrn Vash1 Veph1 Vwf Wwtr1 Yap1 Zeb1 Zhx3 Zfp521 Zfp558
Notch1	0,00	3,22	Abi3 Acvrl1 Angpt2 Arap3 Arnt2 Arrb1 Bcl6b Bicc1 Bmpr1a Bmx Calcrl Cd40 Cdh5 Chst1 Cldn5 Clec14a Cmtm7 Cxcl12 Cxxc5 Daam2 Dennd3 Depdc7 Dock6 Dock9 Ecscr Egfl7 Eif2b2 Enpp2 Entpd1 Erg Esam Fam124b Fam69b Flt4 Fzd7 Gbgt1 Gpc6 Gpr4 Grap Hhip Hspa12a Ica1 Icam2 Igf2bp1 Igfbp5 Inpp5d Jag2 Klhl4 Lrrc8c Lum Lyl1 Map4k2 Mcam Mctp1 Mfng Mmrn1 Mpp4 Mr1 Myct1 N4bp3 Neo1 Nos3 Nrarp Nrg3 Nrgn Pdgfb Pecam1 Pgf Pkn3 Ppm1f Prex1 Prkch Ptpre Rgs12 Robo4 Scarf1 Sema6b Slc31a2 Smad7 Snca Sncaip Sox17 Sox7 St8sia4 Stab1 Sult1b1 Sytl2 Tal1 Tctex1d1 Tie1 Tm4sf1 Tmem44 Tnfrsf10b Tnfrsf11a Trps1 Txnrd2 Vash1 Yap1 Zfp239 Zfp558 Zfp655
Rbpj	0,17	3,22	Abca1 Abi3 Alpk2 Arhgdib Arhgef15 Art4 Atp2b1 Bag2 Calcrl Cd93 Cdh5 Cldn5 Clec14a Clec1a Cnih3 Cps1 Cxcr4 Dnm1 Ecscr Egfl7 Elmo1 Epb41l4a Erg Esam Fam69a Fermt3 Fgd5 Frmd4b Fry Fzd4 Fzd7 Gfra1 Ggt5 Gimap1 Gimap8 Gja4 Hcls1 Hspa12b Hyal2 Ica1 Icam2 Ifi27 Irf6 Irs1 Itga6 Kcnma1 Kdr 2010300C02Rik Kif26a Laptm5 Lyl1 Malt1 Mast4 Mfng Mgat4a Mmrn2 Mpzl2 Myct1 Nlrp2 Nos3 Nrgn Nsun5 Nudt4 Olfml2b Pbx4 Pcdh17 Pdgfra Pecam1 Plcb4 Plekhg1 Plxna2 Podxl Prex1 Prkch Ptger4 Ptp4a2 Ptprb Ptprn2 Rfk Robo4 Rpl37 Runx2 S1pr1 Sdpr Sema3c Sh2d3c Sh3tc1 Shank3 Slc25a3 Sntb1 Sox17 Sox18 Sox7 Srgn St6gal1 St6galnac3 Stab1 Stx11 Tek Trbc1 Trbc2 Trps1 Vwf Zfp185 Nfkb1 Pten
Ifi205	0,14	3,08	A2m Acp5 Adap2 Aif1 Anxa2 Aoah Apobr Apoc1 Apol6 Aqp9 Arhgap18 Asah1 Atp6ap2 C1qa C1qb C1qc C2 C3ar1 C5ar1 Capg Ccl3 Ccl9 Ccl6 Ccr1 Ccr1l1 Ccr2 Ccr5 Cd14 Cd163 Cd300c Cd300c2 Cd300a Cd33 Siglech Cd63 Cd68 Cd86 Cebpa Cebpb Ces1a Cfd Cfp Cldn23 Clec12a Clec4a1 Clec4a3 Clec4a4 Clec4b1 Clec4a2 Clec4b2 Clec4e Clec5a Clec7a Col8a2 Cpvl Crtam Csf1r Csf2ra Csf3r Stfa2l1 Stfa2 Stfa3 Gm5483 BC117090 Gm5689 Stfa1 Gm4758 Csta1 Gm5416 2010005H15Rik BC100530 Ctsh Ctss Cxcl10 Cxcl16 Cxcr2 Cxcr1 Cybb Cyp27a1 Dab2 Dmxl2 Dok3 Dsc2 Egr2 Epb41l3 Fam105a Fbp1 Fcer1g Fcgr1 Fcgr2b Fcgr3 Fcgr4 Fcgrt Fgl2 Fn1 Fpr1 Fpr-rs3 Fpr3 Fpr2 Fpr-rs4 Fpr-rs7 Fpr-rs6 Ftl1-ps1 Ftl1 Fuca2 Glipr2 Gns Gpnmb Gpr173 Gpr34 Gprin3 Grn Havcr2 Hcar2 Hck Hexb Hk3 Hnmt Hpgds Ifi30 Ifit3 Ifit3b Igsf6 Il13ra1 Il18 Il1b Il6ra Irak3 Itgam Itgax Kcne3 Kctd12 Kmo Kynu Lactb Lair1 Lamp1 Lamp2 Lilrb4a Lilra6 Gm14548 Pira2 Pirb Gm15922 Gm15448 Lilra5 Lpl Lst1 Lyz1 Lyz2 9530003J23Rik Mafb Marco Me1 Mfsd1 Mitf Mospd2 Mpeg1 Pfpl Mrc1 Ms4a14 Ms4a4a Ms4a4c Ms4a4b Ms4a4d Ms4a6c Ms4a6b Ms4a6d Ms4a7 Msr1 Mx1 Mx2 Myo1f Ncf2 Nfatc2 Nlrc4 Nod2 Npc2 Npl Nptn Oas1c Oas1e Oas1b Oas1f Oas1h Oas1g Oas1a Oas1d Ogfrl1 Olr1 Oscar P2ry13 Pik3ap1 Pilra Pilrb1 Pilrb2 Pla2g7 Plaur Plbd1 Plxdc2 Ppic Psap Psd3 Ptafr Rab11fip1 Rab31 Rab7b Rassf4 Rbm47 Rnase6 Rnasel Rnf13 Rtn1 S100a8 S100a9 Samhd1 Scarb2 Scpep1 Sema3c Serpina1d Serpina1b Serpina1a Serpina1c Serpina1e Sgms2 Siglec1 Slamf8 Slc15a3 Slc22a15 Slc27a3 Slc31a2 Slc38a6 Slc47a1 Slc7a7 Slco2b1 Snx10 Sod2 Sort1 Spi1 Srrt Stx11 Sucnr1 Tbxas1 Tfec Tgfbi Tlr1 Tlr2 Tlr4 Tlr5 Tlr8 Tm6sf1 Tmem52b Tnfsf13 Tnfsf13b Trem1 Tyrobp Vamp3 Vsig4 Wdfy3 Wls Zfp827

Supplementary Table 4. Analysis of downregulated regulons in stressed *Tph*2$^{+/-}$ vs. wildtype female mice. Acronyms of regulons (columns **A**) refer to gene names of corresponding transcription factors. Involvement of known transcription factors in the genome-wide transcriptional responses to stress on wildtype (column **B**) and mutant (column **C**) backgrounds. Gene targets of the transcription factors are listed in a column **D**. Worksheet 1: stimulated regulons, worksheet B: suppressed regulons. Numbers in the columns B and C are -log10(FDR), where FDR is the false discovery rate associated with the involvement of the respective regulon in the transcriptional response. FDR=0.05 corresponds to the table value of approximately 1.3.

References for Table 1:

1. Wang, C. et al. Methyltransferase-like 21e inhibits 26S proteasome activity to facilitate hypertrophy of type IIb myofibers. The FASEB Journal 33, 9672–9684 (2019).
2. Wang, Y. et al. KDM6B cooperates with Tau and regulates synaptic plasticity and cognition via inducing VGLUT1/2. Mol Psychiatry 27, 5213–5226 (2022).
3. Barnum, S. R. C4b-Binding protein, a regulatory protein of complement. Immunol Res 10, 28–42 (1991).
4. Guo, W. et al. Investigating the expression, effect and tumorigenic pathway of PADI2 in tumors. Onco Targets Ther Volume 10, 1475–1485 (2017).
5. Webster, E. et al. De novo PHIP -predicted deleterious variants are associated with developmental delay, intellectual disability, obesity, and dysmorphic features. Molecular Case Studies 2, a001172 (2016).
6. Bacalini, M. G. et al. Association of rs3027178 polymorphism in the circadian clock gene PER1 with susceptibility to Alzheimer's disease and longevity in an Italian population. Geroscience 44, 881–896 (2022).
7. Strehlow, V. et al. GRIN2A -related disorders: genotype and functional consequence predict phenotype. Brain 142, 80–92 (2019).
8. Tu, H. et al. Up-regulation of golgi α-mannosidase IA and down-regulation of golgi α-mannosidase IC activates unfolded protein response during hepatocarcinogenesis. Hepatol Commun 1, 230–247 (2017).
9. Woerden, G. M. et al. TAOK1 is associated with neurodevelopmental disorder and essential for neuronal maturation and cortical development. Hum Mutat 42, 445–459 (2021).
10. Gong, Y.-T. et al. Advances in the study of the role and molecular mechanism of with no lysine kinase 3 in nervous system diseases (Review). Mol Med Rep 23, 393 (2021).
11. Alkelai, A. et al. A role for TENM1 mutations in congenital general anosmia. Clin Genet 90, 211–219 (2016).
12. Zuo, H. et al. Structural basis for auxiliary subunit KCTD16 regulation of the GABA B receptor. Proceedings of the National Academy of Sciences 116, 8370–8379 (2019).
13. Jullien, P. E. et al. Functional characterization of Arabidopsis ARGONAUTE 3 in reproductive tissues. The Plant Journal 103, 1796–1809 (2020).
14. Anji, A., Hanley, N. R. S., Kumari, M. & Hensler, J. G. The role of protein kinase C in the regulation of serotonin-2A receptor expression. J Neurochem 77, 589–597 (2001).
15. Liu, T., Li, H., Hong, W. & Han, W. Brefeldin A-inhibited guanine nucleotide exchange protein 3 is localized in lysosomes and regulates GABA signaling in hippocampal neurons. J Neurochem 139, 748–756 (2016).
16. Liu, L. et al. Effect of Zbed6 Single-Allele Knockout on the Growth and Development of Skeletal Muscle in Mice. Biology (Basel) 12, 325 (2023).
17. Dines, M. & Lamprecht, R. The Role of Ephs and Ephrins in Memory Formation. International Journal of Neuropsychopharmacology 19, pyv106 (2016).
18. Chmielewska, J. J. et al. PTPN4 germline variants result in aberrant neurodevelopment and growth. Human Genetics and Genomics Advances 2, 100033 (2021).
19. Shen, Z., Wang, X., Yu, X., Zhang, Y. & Qin, L. MMP16 promotes tumor metastasis and indicates poor prognosis in hepatocellular carcinoma. Oncotarget 8, 72197–72204 (2017).
20. Elkins, E. A., Walti, K. A., Newberry, K. E. & Lema, S. C. Identification of an oxytocinase/vasopressinase-like leucyl-cystinyl aminopeptidase (LNPEP) in teleost fish and evidence for hypothalamic mRNA expression linked to behavioral social status. Gen Comp Endocrinol 250, 58–69 (2017).
21. Shook, D., Brouwer, R., de Zeeuw, P., Oranje, B. & Durston, S. XKR4 Gene Effects on Cerebellar Development Are Not Specific to ADHD. Front Cell Neurosci 11, (2017).
22. Strauss, K. A. et al. A population-based study of KCNH7 p.Arg394His and bipolar spectrum disorder. Hum Mol Genet 23, 6395–6406 (2014).
